# Sea-level changes modulate beach face slope in coastal upwelling zones

**DOI:** 10.1038/s41598-026-40630-3

**Published:** 2026-02-20

**Authors:** Marius Aparicio, Laurent Lacaze, Rafael Almar, José M. Alsina

**Affiliations:** 1 Toulouse INP, CNRS, IMFT, University of Toulouse, Toulouse, France; 2 LEGOS (CNRS-IRD-CNES-University of Toulouse), Toulouse, France; 3 Laboratori d’Enginyeria Marítima, Universitat Politècnica de Catalunya, 08034 Barcelona, Spain

**Keywords:** Climate sciences, Natural hazards, Ocean sciences

## Abstract

Although waves are commonly regarded as the primary drivers of beach profile evolution, observations and experiments show that the slope of the beach face does not always directly reflect the conditions of the incident waves. The mechanisms governing transitions between equilibrium states are unclear due to feedback loops and delayed sediment responses. In this study, we analyze 3.5 years of daily beach profile data from two tropical low-tide terrace sites in order to characterize nearshore transient periods between seasonal equilibria. Our results reveal episodes in which the traditional classification of equilibrium beaches fails to predict slope evolution. We demonstrate that, at these beaches, transient events can be influenced by water-level variations linked to coastal upwelling, thereby altering sediment redistribution across the swash–surf continuum. Complementary laboratory experiments confirm that the observed dynamics can be reproduced by considering both the Dean number ($\Omega _0$), representing wave energy, and nearshore water-level variability. Our findings emphasize that transient sea-level modulations, such as those induced by upwelling, mesoscale eddies or atmospheric systems, can reshape beach profiles across multiple timescales. This highlights the need to extend equilibrium frameworks to account for non-wave-driven processes.

## Introduction

The Equilibrium Beach Classification (EBC) for microtidal, wave-dominated environments^1^ is based on a dimensionless number that delineates key equilibrium states. This parameter combines significant wave height ($H_s$), peak wave period ($T_p$), and sediment fall velocity ($w_s$), and is defined as $\Omega _0 = H_s / (T_p w_s)$. The EBC framework consequently identifies two primary transitions: an upstate path, in which the beach evolves from a reflective profile ($\Omega _0 \le 1$) to a fully dissipative profile ($\Omega _0 \ge 6$), and a downstate path in the reverse direction^1,2^. While the EBC provides a robust framework for describing recurring morphological patterns observed on beaches worldwide, it constrains the system to evolve smoothly between *a priori* known equilibrium states. Yet, equilibrium states are not necessarily reached *in situ*, making their definition difficult to assess^2^. Moreover, high-temporal-resolution observations suggest that, even within a single season, multiple equilibrium states may coexist. Transitions between these states are influenced by a myriad of factors, including storm frequency and sequencing^3^, stochastic day-to-day wave forcing, and the fact that each iso-elevation of the profile exhibits its own characteristic timescale toward equilibrium^4^. These factors introduce response lags^5^ and feedback loops inducing semi-annual cycles and asymmetric pathways between accretive and erosive dynamics that further complicate the morphodynamics of the system^5–7^. This has led to the introduction of a site-specific decaying system memory in beach evolution models^8,9^, acknowledging that first-order Markov chain behavior in beach evolution, as proposed by Sonu and James^10^, may only be valid within an idealized closed system where equilibrium states are clearly defined and external variability is minimal. Altogether, these complexities reinforce the need for accurately predicting transitions between stationary configurations to extend the EBC framework. A first approach to tackle this objective is to examine the nearshore (encompassing both swash-surf continuum) transients in order to delineate all the parameters of interest to describe their dynamics.

In this context, the swash zone, defined as the region of wave run-up and run-down around the mean water level, is a good candidate for highlighting the trends of transients in beach evolution. Its steepness, referred to as the beach face slope ($\beta$), directly influences the swash zone’s capacity to reflect incident wave energy. According to the EBC framework, the beach face slope is expected to flatten during upstate transitions (as $\Omega _0$, or offshore wave energy, increases) and steepen during downstate transitions (as $\Omega _0$ decreases). A comprehensive dataset of beach face slope measurements^11^ (spanning sediment sizes from fine sand to boulders) was used to test various equilibrium formulations, assuming that the equilibrium steepness of the beach face is governed by both grain characteristics (size, density, and porosity) and wave parameters (height, period, and steepness) in microtidal environments^12–14^. While these formulations show promising predictive skill, they typically assume a linear or power-law dependence of $\beta$ on mean grain size and wave parameters, yet fail to fully capture the broad range of observed slopes for a given sediment size. These contradictions stem from two assumptions that do not hold in systems continuously forced at their boundaries: (1) $\beta$ is assumed to be in instantaneous equilibrium with incoming wave conditions. Although the beach face slope may respond rapidly to forcing (over a few hundred to a few thousand waves), it still exhibits lags and the temporal scale for which the equilibrium assumption holds true can only be deduced $\textit{a posteriori}$ from observations. (2) $\beta$ is assumed to adapt solely by rotating around the shoreline, focusing exclusively on sediment exchanges between the lower and upper portions of the slope. This has led to the classical framework in which onshore sediment transport steepens the slope while offshore transport flattens it^15^, an oversimplification that neglects complex sediment redistribution patterns during profile adjustment.

**Fig. 1:**
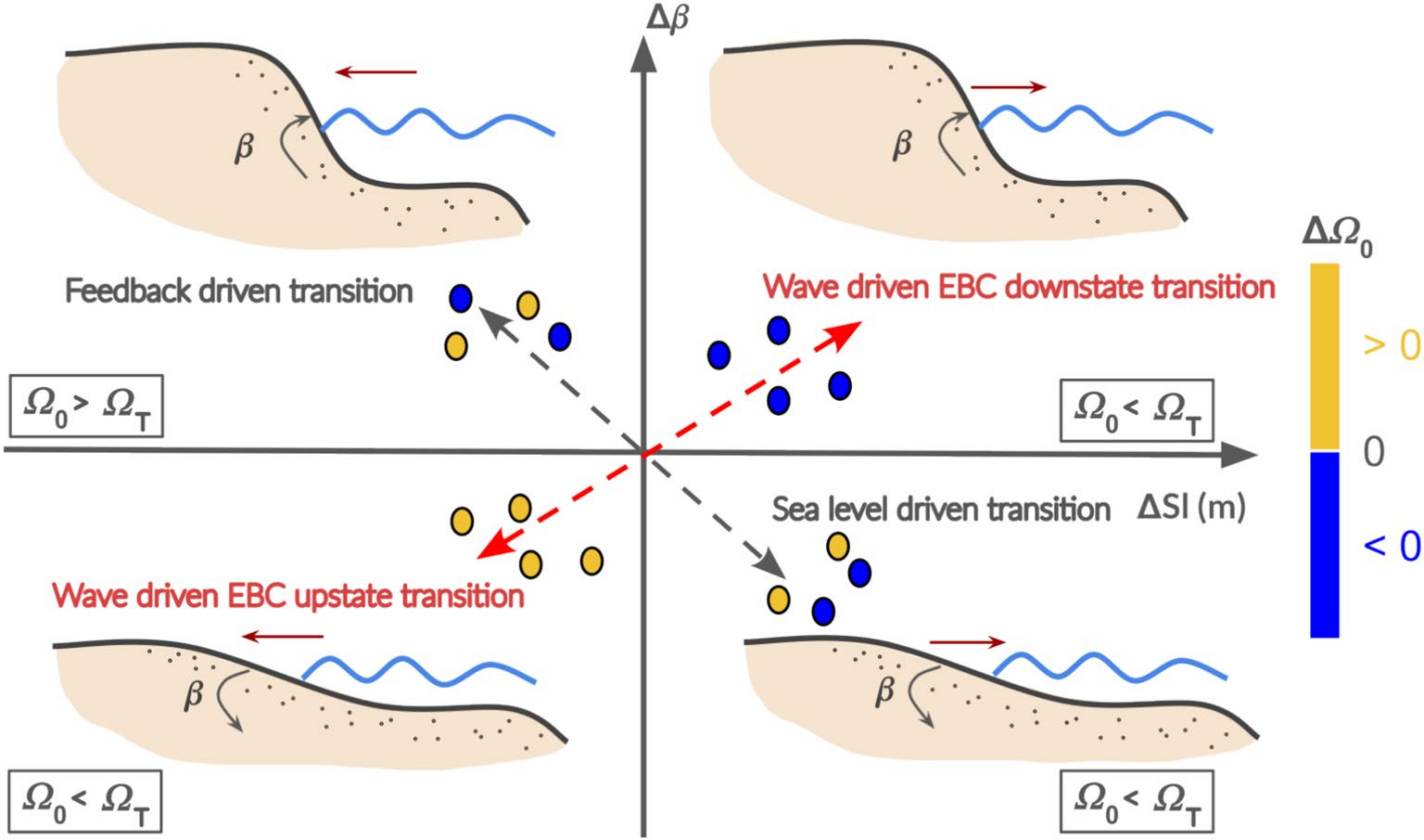
Schematic representation of the Swash Dynamic Diagram. Quadrant points represent time intervals during which both $\beta$ and *Sl* are changing, while the origin denotes equilibrium conditions for both parameters. Points along the axes indicate periods where either $\beta$ or *Sl* individually reaches equilibrium: slope translation occurs along the x-axis, and direct slope adaptation to wave forcing without sediment transport influence occurs along the y-axis. The beach illustrations depict nearshore cross-shore profiles typical of low Dean number environments (reflective to LTT), where the surf and swash zones are distinguishable. The EBC-predicted coevolution mode, related to the sign of $\Delta \Omega _0$, is indicated by the dashed-red arrow. A second coevolution mode, related to feedback and sea level modulations, represented by the gray-dashed arrow, has been observed in the field but lacks a clear and consistent dependence on the sign of $\Delta \Omega _0$. The LTT sub-classification SwRB regime ($\Omega _0 < \Omega _T$) and SRB ($\Omega _0 > \Omega _T$) associated to each quadrant are reported accordingly to field observations.

During transients, $\beta$ should be considered an integral component of the nearshore continuum, influenced by sand exchanges between the surf and swash zones. In such a context, a correlation between shoreline displacement and the slope’s trajectory toward equilibrium might be expected, yet this relationship remains poorly understood. Nevertheless, nearshore evolution through erosion and accretion involves cross-shore sediment transfers between the aforementioned zones, which likely influence slope adjustment independently of the direct effects of incident wave forcing. Accordingly, to fully capture swash zone dynamics, it is essential to consider both the changes in shoreline position and beachface slope steepness. For this purpose, the Swash Dynamic Diagram (SDD) framework is introduced to illustrate the coevolution of the beach face slope and the shoreline position anomaly, defined as the deviation from its mean value over the monitoring period (*Sl*), as they evolve toward a full equilibrium (Fig. 1). In this framework, the temporal variation of $\beta$ ($\Delta \beta$) is analyzed as a function of the cross-shore sediment transport variation, represented by changes in *Sl* ($\Delta Sl$). Using this representation, the wave driven EBC downstate and upstate transitions can be visualized. Downstate transitions scatter into the upper right quadrant ($\Delta Sl > 0$, $\Delta \beta > 0$) for $\Delta \Omega _0 < 0$, where shoreline progradation steepens the slope. Conversely, upstate transitions appear in the lower left quadrant ($\Delta Sl < 0$, $\Delta \beta < 0$) for $\Delta \Omega _0 > 0$, where shoreline retreat flattens the slope (red double arrow in Fig. 1).

Be that as it may, the field observations of transient beach profiles evolving toward seasonal equilibrium conducted in this study revealed the emergence of a second mode of coevolution between $\beta$ and *Sl*. This mode does not display a clear or consistent correlation with the sign change of $\Delta \Omega _0$, particularly under low wave energy conditions. In this second mode, the beach face slope steepens during erosion periods ($\Delta Sl < 0$, $\Delta \beta > 0$), with data points clustering in the upper left quadrant. Conversely, during accretion periods, the slope flattens ($\Delta Sl > 0$, $\Delta \beta < 0$), with points falling into the lower right quadrant (gray double arrow in Fig.  1). The emergence of this second mode challenges the traditional understanding of the EBC’s adjustment pathways, emphasizing the need to reconsider the parameters driving trajectory bifurcation during transient periods.

The recent field study by Mingo et al.,^16^ investigated the relationship between $\beta$ and $\Omega _0$ in Low Tide Terrace (LTT) environments, commonly characterized by a shallow, submerged sandy terrace^1^. They identified discrepancies between the expected response of $\beta$ to $\Omega _0$, as predicted by the equilibrium beach classification, and actual observations. Notably, a shift in the correlation between $\beta$ and $\Omega _0$ was observed around a threshold $\Omega _T \sim 2.5$. This led to the proposal of an LTT sub-classification based on the role of the sandy terrace in wave dissipation, distinguishing two regulation regimes: swash-regulated beaches (SwRB) and surf-regulated beaches (SRB). Below the $\Omega _T$ threshold, LTTs are classified as SwRB. In this regime, $\beta$ decreases as $\Omega _0$ increases, with the beach face slope rapidly adapting to wave energy, consistent with EBC state transitions. Above the $\Omega _T$ threshold, LTTs switch to SRB, where the sandy terrace significantly influences wave energy dissipation. In this regime, $\beta$ is not well-defined at a given $\Omega _0$ and may even increase along with $\Omega _0$, making LTTs profile a turning point between reflective and other intermediate states (toward dissipative). More recently, Mingo et al.,^17^ experimentally demonstrated the necessity of introducing a local Dean number, $\Omega _{swash} = H_b / (T_p w_s)$, to better predict the beach face slope equilibrium steepness. Here, $H_b$ represents the bore height entering the swash zone, derived from an extrapolation of bore height attenuation across the surf zone. This local Dean number accounts for the surf zone’s influence on the nearshore dynamics, capturing the shift in offshore $\Omega _0$ at the shore. These findings emphasize the critical role of the surf zone in beach face slope adaptation, demonstrating that $\beta$ cannot be solely linked to the offshore wave forcing via $\Omega _0$ because of feedback effects. This sub-classification will be developed here and used to analyze transient periods.

In the following, we found interest in the nearshore of LTT environments ($\Omega _0 \simeq$ 2)^2^ and for whom surf-swash interplay is strong^18^, in order to unravel transient periods between seasonal equilibria and confront their dynamics to the EBC framework predictions. The presence of a quasi-periodic coastal upwelling suggested that water level modulations could be a key driver of transient periods beyond the scope of the EBC. This hypothesis was tested using a laboratory experimental setup scaled to qualitatively replicate LTT environments. The experiments successfully reproduced all transient dynamics observed in the field due to the interplay between $\Omega _0$ and the water level, indicating the potential role of coastal processes, through water level modulations, in shaping beach morphology across multiple timescales.

## Results

### Ground-based data sets

**Fig. 2:**
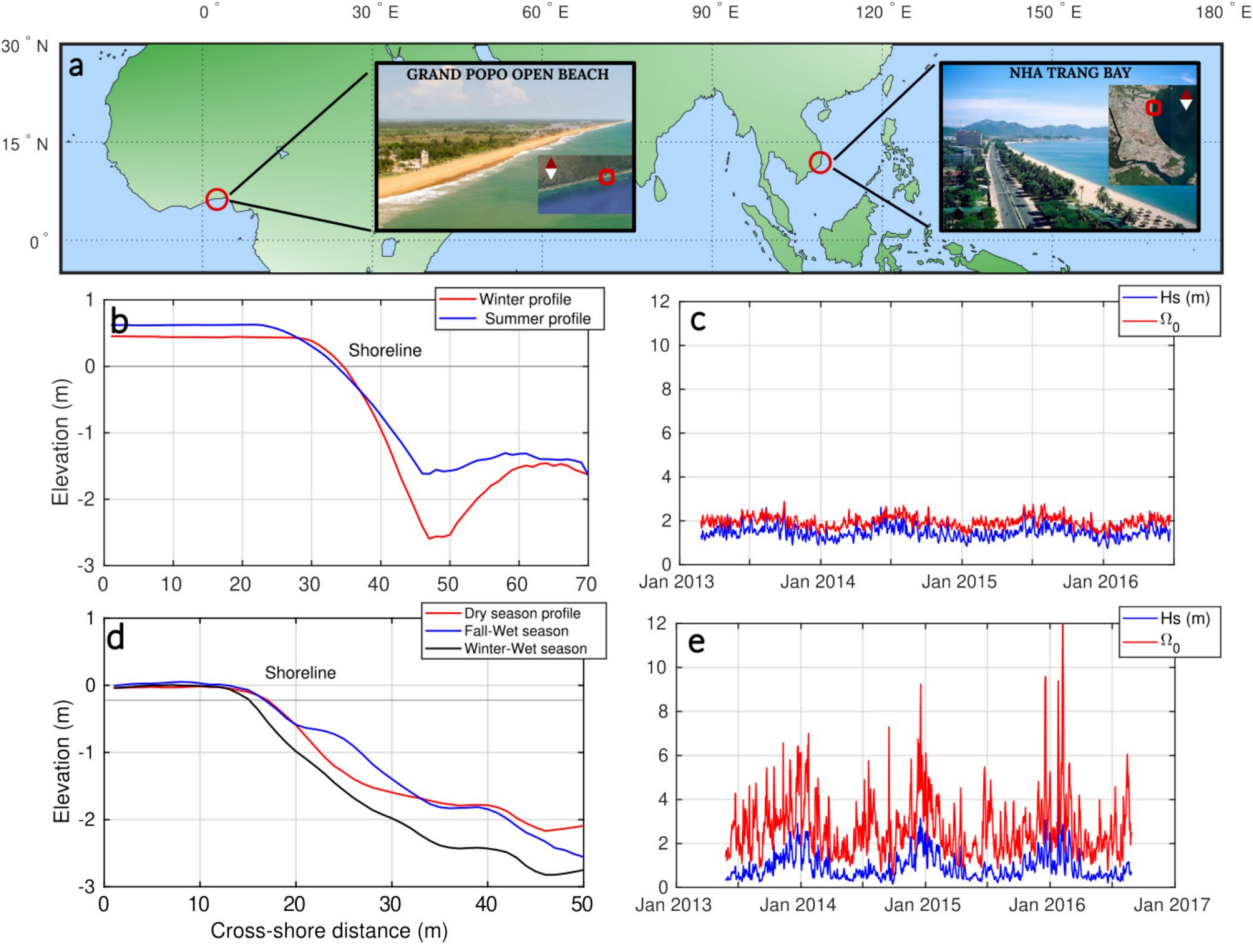
Overview of the Study Sites’ Characteristics. (**a**) Grand Popo is an open beach located in Benin, West Africa. The beach, facing south toward the Atlantic Ocean, is exposed to a relatively constant southerly swell. Nha Trang is an embayed beach in southern Vietnam, subject to seasonal variations in wave climate, both in intensity and direction, driven by the monsoon. Both sites exhibit microtidal low-tide terrace (LTT) profiles, characteristic of low average $\Omega _0$ conditions. The tidal range at Grand Popo varies from 0.8 m to 1.8 m, and from 0.4 m to a maximum of 1.7 m at Nha Trang. Red squares overlaid on satellite images indicate the exact monitoring areas at each site.(**b**,**c**) Seasonal equilibrium profiles and daily-resolved wave forcing time series for Grand Popo. (**d**,**e**) Equivalent plots for Nha Trang. These panels highlight key differences in wave exposure: Grand Popo’s seasonal LTT profiles are modulated by a relatively stable, low-amplitude seasonal signal, whereas Nha Trang experiences a more variable wave climate, with storms punctuating the seasonal modulation. For both sites, breaker significant wave height and peak period were extracted from pixel intensity thresholds, with missing video data reconstructed via linear regression against Wavewatch III v4.10 (Grand Popo) and ERA-Interim reanalysis (Nha Trang); more details in Methods). Photo credits: Sampo Kiviniemi and Cami Marty.

The two data sets used in this study have been acquired using a shore-based video system over 3.5 years with daily resolution (Methods). Both sites are wave-dominated, micro-tidal environments with similar mean sediment size ($D_{50}$ = 0.6 mm) and sediment fall velocity ($w_s$ = 0.084,m $\cdot$ $s^{-1}$)^16^. These sites are identified as LTTs in the equilibrium beach classification (Fig. 2b,d & Methods), and they exhibit a reflective upper profile (steep slope) and a dissipative lower profile, including a sandy terrace that can significantly influence wave transformation and energy dissipation, depending on wave conditions and tidal range. Moreover, in the classification proposed by Bujan et al.^11^ where $\beta (D_{50})$, the typical steepness range of these beaches lies between 0.07 and 0.2, as also found by Mingo et al.^16^. However, significant differences in their morphodynamic evolution suggest the need for a more nuanced classification.

Grand Popo (Fig. 2a–c) is an open beach exposed to a relatively low energetic swell generated in extra-tropical regions, consistent all along the year ($\overline{H_s}$ = 1.5 m, $\overline{T_p}$ = 8.9 s). The corresponding annual average Dean number $\overline{\Omega _0}\approx 2$ is modulated by a low amplitude seasonal signal. The sedimentary cell including Grand Popo is exposed to one of the highest rates of longshore drift in the world^19^ and the beach consistently falls within the SwRB regime^16^, meaning that most of the incoming wave energy is handled by the swash zone. The beach face slope adapts to the wave forcing, mostly following the EBC, i.e. increasing wave energy (or equivalently $\Omega _0$) results in lowering the beach face slope and vice versa^16^. In addition, the water level in the surf zone (i.e. above the sandy terrace) is quite high ($\overline{h_t}$ = 1.7 m; i.e. $\overline{H_s} \approx \overline{h_t}$), reflecting a weakly active terrace during wave transformation.

Nha Trang (Fig. 2a,d,e) is an embayed beach located in south Vietnam, experiencing a tropical climate with a wet season from October to April, where typhoons are expected, and a dry season from May to September. During the wet season, two successive wave regimes occur^20–22^: (1) from October to December (namely fall-wet season), an energetic northeast directed swell surges at the coast ($\overline{H_s}$ = 1.5 m, $\overline{T_p}$ = 5 s, $\overline{\Omega _0}\approx 3.5$) and (2) from January to the end of April (namely winter-wet season), a low energetic regime slowly takes place ($\overline{H_s}$ = 0.8 m, $\overline{T_p}$ = 4.4 s, $\overline{\Omega _0}\approx 2.2$). Then, from May to September the dry season induces a change in wave direction and a gentle southeast directed swell dominates the wave conditions ($\overline{H_s}$ = 0.6 m, $\overline{T_p}$ = 3 s, $\overline{\Omega _0}\approx 2.4$)^23^. According to Mingo et al.,^16^ Nha Trang can therefore be SwRB during the dry season or SRB during the wet season. Finally, the water level over the terrace varies more significantly during the year than that of Grand Popo (see seasonal profiles in Fig. 2b,d). Its average value over the year is $\overline{h_t} = 0.75\,$m, meaning that wave climate $\overline{H_s}$ can be significantly affected by the sandy terrace depending on the season.

In the following, the swash zone evolution at both reference sites is characterized by its beach face slope angle $\beta$, defined in Mingo et al.,^16^ as the linear portion between the berm crest and the upper limit of the terrace, and the shoreline position anomaly $Sl = x_s(t)-S$, with $S=\overline{x_s(t)}$ the average cross-shore position of the shoreline over the entire data set. The sensitivity of $\beta$ to uncertainties in profile position estimations was assessed using Monte Carlo statistical tests^24^, revealing no significant changes in either trend or value (see Methods).

### Beach transients

**Fig. 3:**
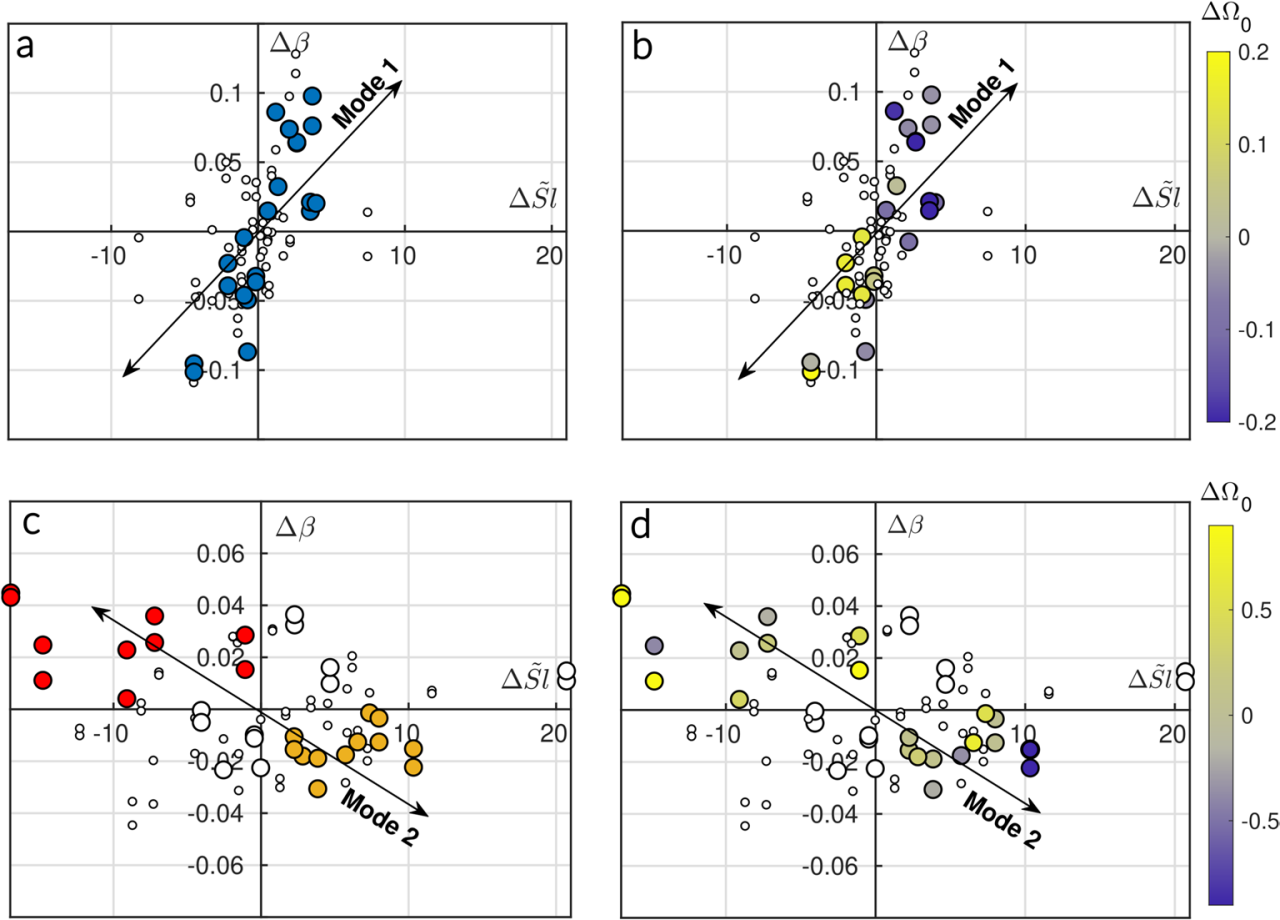
Field data projection in the SDD. Temporal variations of $\beta$ ($\Delta \beta$) as a function of temporal variations of the $\tilde{Sl}$ ($\Delta \tilde{Sl}$) for Grand Popo (**a**, **b**) and Nha Trang (**c**, **d**). (**a**, **c**) Transients identified at both beaches (Methods) are highlighted by the large dots with the similar color code (blue, orange and red), while the periods outside the transients are represented by small white dots. For Nha Trang (**c**,**d**), transients falling under Mode 1 are depicted by large white dots to ease readability. (**b**, **d**) The same plots are shown with the transition periods along with the color map of their corresponding $\Delta \Omega _0$. The $\Delta$ time step is one month. The samples have been densified by running monthly $\Delta$ starting at different times in the month to emphasize the strength of the mechanism. Solid black double arrows serve as visual guides for highlighting co-evolution modes only.

Transients are defined based on morphological variations of the beach profile between seasonal equilibrium states (Methods). Accordingly, the temporal variation of the monthly averaged beach face slope, $\Delta \beta$, is analyzed as a function of the shoreline position change, $\Delta Sl$, within the framework of the SDD diagram (Fig. 3). From this point onward, only dimensionless variables and their associated physical processes are used to enable direct comparison between field sites and experimental models at the laboratory scale (Methods). In this context, *Sl* is redefined as a dimensionless shoreline position: $\tilde{Sl} = (x_s(t) - S)/h_t$, where $h_t$ represents the water level over the sandy terrace.

Grand Popo’s swash zone is characterized by a single mechanism of coevolution under a SwRB regime ($\Omega _0 < \Omega _T$), following the wave-driven transitions defined by the EBC framework. In this regime, accretion (erosion) consistently corresponds to an increase (decrease) in $\beta$ (Fig. 3a). These transitions are referred to as Grand Popo Winter (GPP-W) and Grand Popo Summer (GPP-S) transients, respectively (Table 1, Fig. 10). Figure 3b shows that this mode of coevolution (Mode 1) results directly from the beach face slope responding to offshore wave energy. Most points within the frame $\Delta \beta > 0$, $\Delta \tilde{Sl} > 0$ are associated with a decrease in $\Omega _0$, while points in the $\Delta \beta < 0$, $\Delta \tilde{Sl} < 0$ frame align with an increase in $\Omega _0$. However, this relationship is not universally valid as some events with large $\Delta \Omega _0$ do not produce significant changes in $\tilde{Sl}$ or $\beta$, which may involve hysteresis effects, as previously reported in controlled laboratory experiments^5^.

In contrast, Nha Trang’s swash zone exhibits two distinct modes of coevolution (Fig. 3c). The first mode resembles that observed at Grand Popo under a SwRB regime and is associated with transients occurring during the winter-wet season. We refer to these as Nha Trang Winter Wet (NT-WW) transients. The second mode (Mode 2), however, displays an inverted behavior. In this case, transients identified during the fall-wet season – labeled Nha Trang Fall Wet (NT-FW) – are located in the upper left quadrant, where erosion coincides with an increase in $\beta$. These NT-FW transients occur under SRB conditions ($\Omega _0 > \Omega _T$) and are typically observed between September and December, when the wave climate enters its monsoon-driven wet season (Fig. 11). Surprisingly, other transients belonging to Mode 2 – referred to as Nha Trang Dry (NT-D) transients – fall within the lower right quadrant, where accretion is associated with a decrease in $\beta$ despite SwRB conditions ($\Omega _0 < \Omega _T$) (Fig. 11, Table 1). These transients typically take place between May and July, when the wave incidence shifts from north to south due to changes in surface wind patterns. Moreover, Fig. 3|d shows that, unlike Mode 1, Mode 2 cannot be interpreted solely as a response to offshore wave energy, in particular for the lower quadrant of this coevolution mode (NT-D transients). Table 1 further reinforces this interpretation by showing the variations of $\Omega _0$, denoted $\Delta \Omega _0$, between both ends of each transient, revealing that, unlike all other transients, NT-D transients are not triggered by significant changes in $\Omega _0$, as $\Delta \Omega _0 \sim 0$, and that the sign of these variations is not as consistent as those observed in other transients, despite triggering similar morphological evolution on a yearly basis (Fig. 11).

The fact that transients from both SwRB and SRB regimes belong to the same coevolution pattern (Mode 2) challenges the assumption that wave energy alone governs beach face slope dynamics. Given the current state of knowledge, the emergence of the upper quadrant in Mode 2 can be attributed to the enhanced influence of beach morphology on wave transformation (i.e. feedback driven transition in Fig. 1), which, although present in all beach states, becomes dominant under SRB conditions compared to SwRB ones in LTT environments, leading to a decorrelation between $\Omega _0$ and $\beta$, as demonstrated by Mingo et al.^16,17^. Nonetheless, both Mode 1 and the lower quadrant of Mode 2 at Nha Trang occur under SwRB conditions, with largely similar values of $\overline{\Omega _0}$ (Table 1), yet they follow distinctly different coevolution pathways. This indicates that beach face slope adaptation is not solely a response to variations in $\Omega _0$, and that at least one additional parameter–beyond $\Omega _0$–governs swash zone dynamics by controlling sediment redistribution along the beach face slope and, therefore, its trajectory toward equilibrium.

**Table 1:** Recap table of transients characteristics at Nha Trang and Grand Popo.

Nha Trang										Grand Popo	
Transients	NT-FW1	NT-FW2	NT-FW3	NT-WW1	NT-WW2	NT-WW3	NT-D1	NT-D2	NT-D3	GPP-S	GPP-W
$\overline{\Omega _0}$	3.3	3.2	3.0	2.5	1.9	2.3	1.7	2.4	2.4	2	1.9

Mode	SRB	SRB	SRB	SwRB	SwRB	SwRB	SwRB	SwRB	SwRB	SwRB	SwRB
$\Delta \Omega _0$	0.7	1.8	1.3	−1.5	−0.7	−1.8	0.1	−0.1	0.5	0.3	−0.6
$\Delta \beta$	$\mathbf{> 0}$	$\mathbf{> 0}$	$\mathbf{> 0}$	$\mathbf{> 0}$	$\mathbf{> 0}$	$\mathbf{> 0}$	${ < 0}$	${ < 0}$	${ < 0}$	${ < 0}$	$\mathbf{> 0}$
$\Delta Sl$	${ < 0}$	${ < 0}$	${ < 0}$	$\mathbf{> 0}$	$\mathbf{> 0}$	$\mathbf{> 0}$	$\mathbf{> 0}$	$\mathbf{> 0}$	$\mathbf{> 0}$	${ < 0}$	$\mathbf{> 0}$
EBC agreement	$\times$	$\times$	$\times$	$\checkmark$	$\checkmark$	$\checkmark$	$\times$	$\times$	$\times$	$\checkmark$	$\checkmark$

Transients are labeled according to their distinct seasons and years of occurrence (numbers) (see Methods, Figs. 9 and 10). Period-averaged $\Omega _0$, LTT regime, Dean number, slope and shoreline trends, and comparison with the EBC are compiled here. The $\beta$ and *Sl* trends are characterized by the sign of $\Delta \beta$ and $\Delta Sl$ respectively, with color association: blue for $\Delta > 0$ and red for $\Delta < 0$.

### Remaining unexplained transient : the concomitant coastal upwelling

**Fig. 4:**
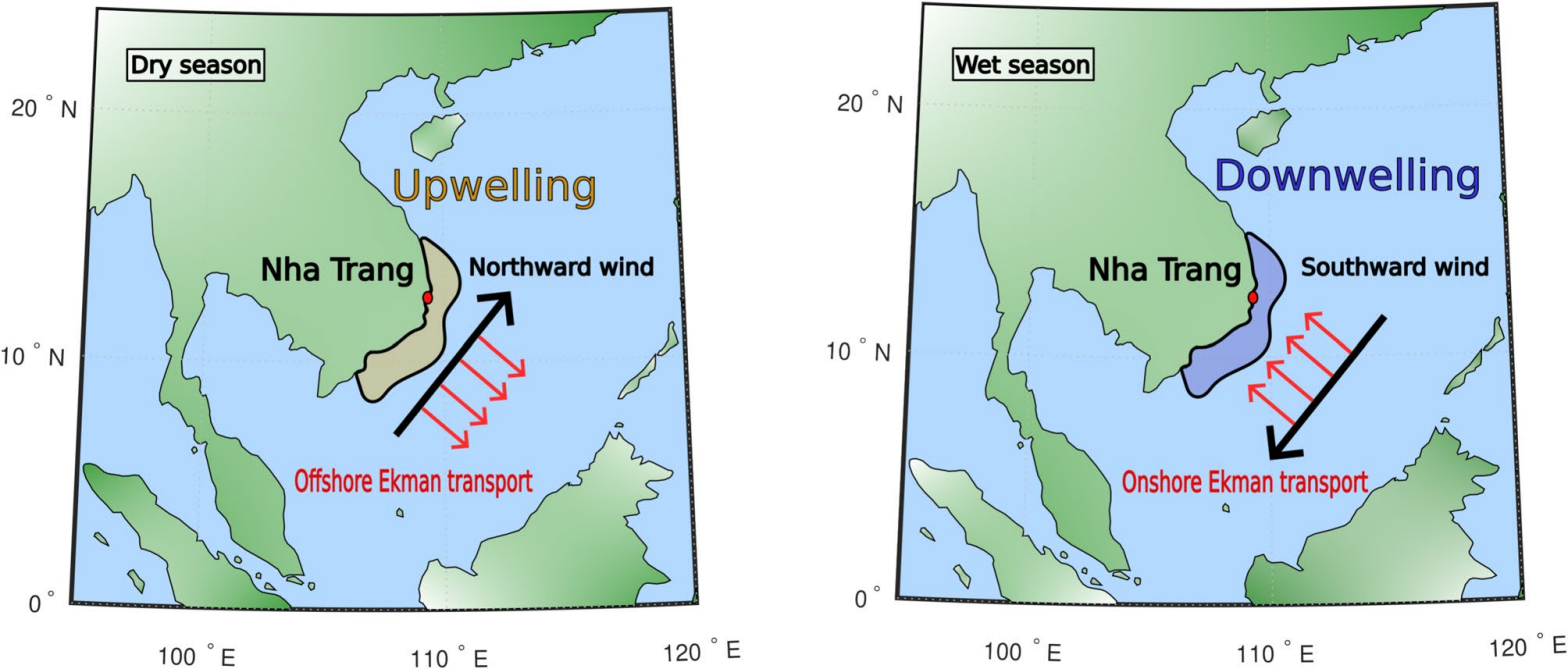
Schematic view of the seasonal change in surface wind direction inducing coastal up/downwelling dynamics. The panels illustrate the seasonal modulation of surface wind direction (black arrows) between the wet season (southward winds) and the dry season (northward winds). The resulting Ekman transports (red arrows), driven by winds and Coriolis effect, lead to coastal downwelling (wet season) or upwelling (dry season) in the nearshore zone (colored regions) due to pressure gradients induced by water redistribution. These processes influence nutrient distribution, surface water levels, and temperature throughout the water column.

**Fig. 5:**
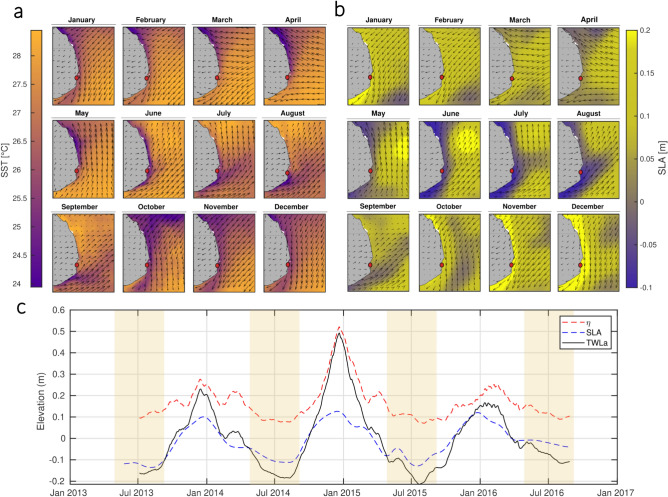
Satellite observations of coastal water conditions near Nha Trang. (**a**) Monthly climatology of sea surface temperature (SST) observed during the monitoring period (2013–2016), with surface wind direction overlaid (black arrows). The maps highlight a seasonal shift in wind direction from May to September, coinciding with a strong SST gradient along the south-central coast of Vietnam. Colder coastal waters relative to offshore regions indicate the ascent of bottom water associated with coastal upwelling. (**b**) Monthly climatology of sea level anomaly (SLA) over the same period, also with overlaid wind direction (black arrows). These maps confirm the presence of upwelling during the wet season (May–September), as evidenced by negative SLA near the coast, indicative of offshore flux divergence driven by upwelling dynamics. Nha Trang location is highlighted by the red dot. (**c**) Time series of wave setup ($\eta$), sea level anomaly (SLA), and total water level anomaly (TWLa) over the beach monitoring period. Dry seasons are highlighted by light-orange shading. This plot illustrates the strong seasonal modulation of water levels at Nha Trang, driven by the alternation between upwelling and downwelling. The dry season is characterized by the lowest TWLa values.

At Nha Trang, the remaining unexplained transient occurs quasi-periodically each year from May up to September. Since beach response may be delayed relative to changes in wave forcing, and that the duration of each transient may vary annually depending on the internal variability of the system (Fig. 11), we simplify its characterization by defining the onset of the dry season as the start of the NT-D transient period. At this point, surface wind direction shifts from southward to northward along the coast, triggering offshore-directed Ekman transport due to the combined influence of surface winds and the Coriolis effect^25^ (Fig. 4). This transport generates a pressure gradient from the seabed toward the surface, driving the ascent of cold, nutrient-rich bottom water^26^. This upwelling process leads to a drop in sea surface temperature (SST) (Fig. 5a), while the associated flux divergence at the sea-air interface results in a negative sea level anomaly (SLA), as observed through satellite altimetry (Fig. 5b). The presence of this seasonal upwelling system along the south-central coast of Vietnam has been confirmed by Kunzmann et al.^27^ through in situ measurements conducted in Nha Trang’s bay.

A closer examination of the water level components time series (Fig. 5c) reveals that the dry season is characterized by strongly negative satellite-derived SLA^28^. Additionally, the wave setup ($\eta$), derived using the semi-empirical formulation from Stockdon et al.,^29^, indicates that the contribution of wave propagation to nearshore water levels (from the breakpoint to the shoreline) decreases during the dry season (Methods). This reduction is primarily attributed to a decline in both incident wave energy and beach slope steepness. Finally, the Total Water Level (TWL) is computed as the sum of wave setup ($\eta$) and sea level anomaly (SLA), and its corresponding anomaly (TWLa) is derived to provide a first-order assessment of water level modulation over the Nha Trang beach profiles (Fig. 5c). This approach captures the combined influence of both wave forcing and coastal upwelling on nearshore water levels and shows minima during each dry season.

The collection of these evidences suggests that the remaining unexplained transient observed during the dry season under SwRB regime, associated with coevolution Mode 2 (Fig. 3c), may be driven by nearshore water level modulations induced by coastal upwelling occurring concurrently with the NT-D transient period. To test this hypothesis, the influence of water level is investigated in a controlled laboratory environment designed to qualitatively replicate the behavior of LTT’s nearshore systems, incorporating both swash and surf zones.

### Physical model: laboratory experiments

To model transient periods between established seasonal equilibrium profiles associated with significant wave regime changes, a laboratory-scale physical model consisting of an idealized sandy beach – comprising an initial sandy terrace ($L^i_{surf} \sim 0.25\,$m) followed by an initial swash slope ($\beta _i \sim 0.2$) – is used (Methods). For each experiment, the beach is exposed to a given wave regime ($Wr_n$) until nearshore equilibrium, i.e. time-invariant slope and shoreline position, is reached. The profile is then exposed to a slightly more energetic wave regime ($Wr_{n+1}$) for twenty minutes before returning to the initial $Wr_n$ until the former equilibrium state is reached again. Four different wave forcings $(Wr_1, Wr_2, Wr_3, Wr_4)$, corresponding to $\Omega _0 = \{1, 1.4, 2, 2.6\}$, are tested in the present set of experiments in order to explore both SwRB and SRB regimes (see Table 3). Furthermore, the same experiments are repeated for two different initial water levels over the terrace, $h_t = \{4 \ \text {cm}, 5\ \text {cm}\}$ – associated to various height ratios $\gamma$ (Table 5 & Methods) – to simulate the water level difference observed at Nha Trang during the coastal upwelling and to test its influence on the dynamics of cross-shore sediment transport. The details of the six runs performed are summarized in Table 2.

**Fig. 6:**
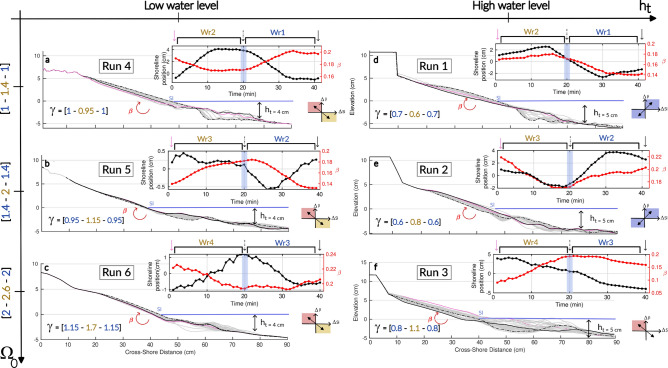
Comprehensive evolution of nearshore beach profiles during laboratory experiments. (**a**–**f**) Results from Runs 1 to 6. Equilibrium profiles for $Wr_n$ at 40 minutes (solid black lines) and $Wr_{n+1}$ at 20 minutes (dash-dotted black lines) are shown alongside their corresponding transient profiles (gray lines). Starting profiles, corresponding to the equilibrium state of $Wr_n$ at 0 minutes, are also displayed (solid pink lines). Insert display the temporal evolution of shoreline position and $\beta$. The light-blue bar indicates the imposed change from $Wr_{n+1}$ to $Wr_{n}$ after 20 minutes of experimentation. Arrows indicate the corresponding timestamp of the associated equilibrium profile, according to their line styles. The dimensionless numbers of control are displayed with a color association to $Wr_{n}$ (blue) and $Wr_{n+1}$ (orange). Beach face slope values are calculated between 0 cm and +2 cm elevation. Sub-panels indicate the coevolution mode followed by the beach in each run. Measurement uncertainties for shoreline position and $\beta$ are 0.9 mm and $5 \times 10^{-3}$, respectively.

**Fig. 7:**
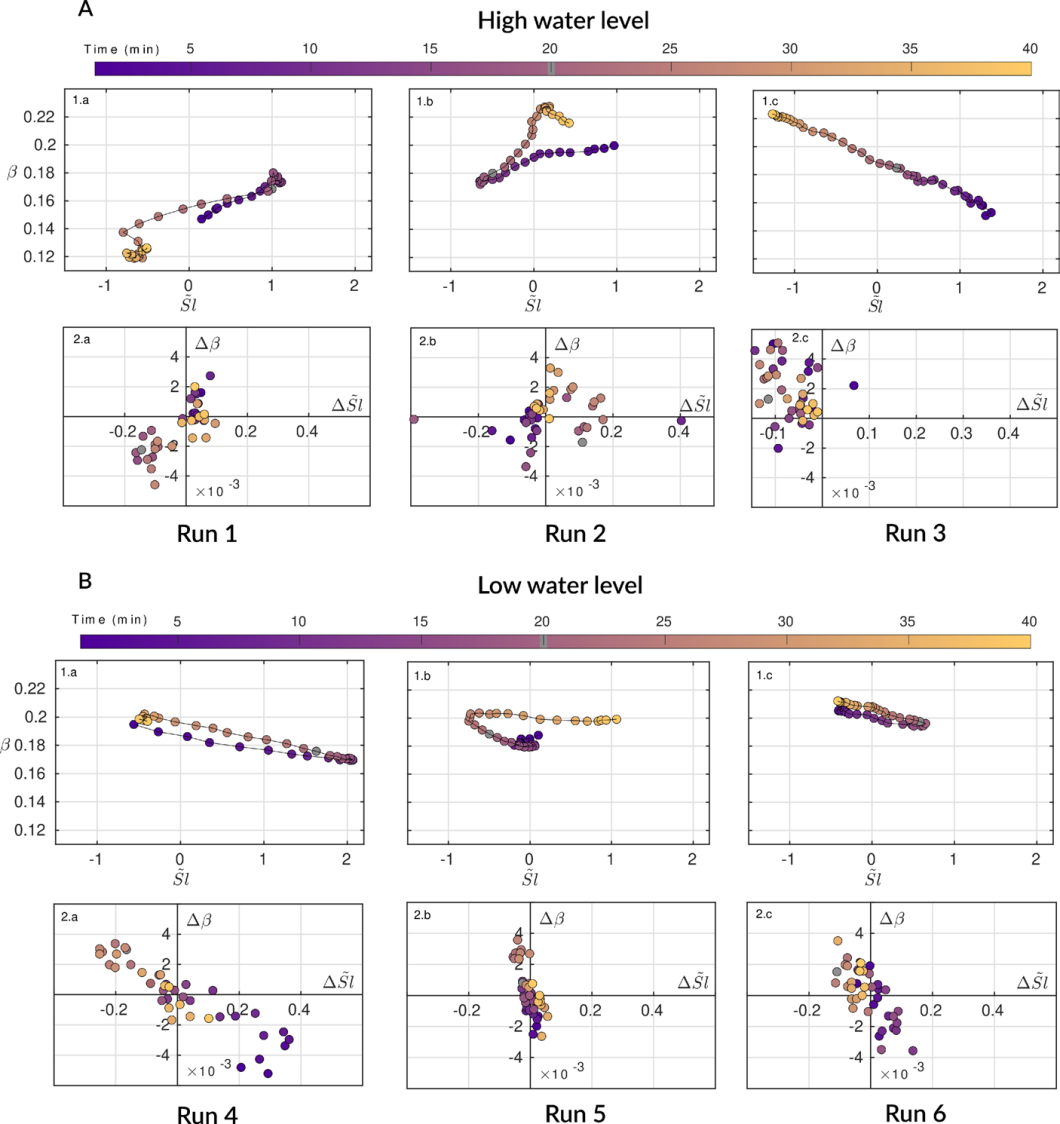
Parameter space and experimental data projection in the SDD framework. (**A**) Experimental runs conducted under high terrace water level condition ($h_t$ = 5 cm): RUN 1–3. (A.1.a, A.1.b, A.1.c) Temporal evolution of the swash zone in the parameter space $(\beta ,\tilde{Sl})$. (A.2.a, A.2.b, A.2.c) Swash Dynamic Diagram ($\Delta \beta$ as a function of $\Delta \tilde{Sl}$). (**B**) Experimental runs conducted under low terrace water level condition ($h_t$ = 4 cm): RUN 4–6. (B.1.a, B.1.b, B.1.c) Temporal evolution of the swash zone in the parameter space $(\beta ,\tilde{Sl})$. (B.2.a, B.2.b, B.2.c) Swash Dynamic Diagram ($\Delta \beta$ as a function of $\Delta \tilde{Sl}$). For each run, between 0 and 20 min (purple color), the beach is exposed to $Wr_{n+1}$. For the remaining run time (20–40 min), the beach is exposed to $Wr_n$ (orange color). The exact timing of the wave regime change is highlighted by the gray insert in the colormap.

Figure 6d–f displays the beach profile evolution for high water level conditions experiments. Together with Fig. 7A presenting the $\beta$-$\tilde{Sl}$ coevolutions in the SDD, we are able to get the full picture of the nearshore transients trajectories. Under SwRB conditions (Run 1-2), the swash zone follows coevolution Mode 1, where accretion and erosion (as well as changes in $\beta$) are driven by variations in $\Omega _0$ (Figs. 6d,e and 7A1.a–b,A2.a–b). Conversely, when the system transitions to a SRB regime (Run 3), the swash zone instead follows coevolution Mode 2 ($\Omega _0 > \Omega _T$), in agreement with the LTT sub-classification threshold^16,17^, before switching back to Mode 1 for whom $Wr_3$ drives beach back under SwRB regimes (Figs. 6f and 7A1.c,A2.c). It is worth noting that in RUN 3, shoreline position and profile evolution show that once the forcing returned to $\Omega _0 < \Omega _T$, the beach did not accrete back. This may indicate inertia in beach response, suggesting that a longer wave exposure would be required for the beach to respond to the forcing reversal. Overall, the high water level experiments demonstrate that the beach face slope steepness evolve coherently toward equilibrium under SwRB conditions (Mode 1). In contrast, SRB conditions produce the upper quadrant of Mode 2 coevolution, in line with the LTT sub-classification and the wave and water level conditions observed at both field sites during the modeled transients.

The impact of negative SLA associated with coastal upwelling at Nha Trang is simulated in the low water level condition experiments (Figs. 6a–c and 7B). In each run (Run 4–6), the swash zone follows coevolution Mode 2, characterized by opposing evolution of $\beta$ and $\tilde{Sl}$ as they move toward their respective equilibrium. This behavior is observed under both SwRB and SRB regimes, indicating that water level conditions control the preferential elevations of accretion and erosion. Consequently, for low water level conditions, the adaptation of the beach face slope appears to be governed not solely by changes in $\Omega _0$, but also by vertical shifts in sediment redistribution driven by water level modulation. Looking closely at the height ratio values, a threshold in $\gamma$ seems to arise in the laboratory experiments around unity. Runs 1 and 2, as well as the SwRB part of Run 3, all present $\gamma < 1$, suggesting weak surf zone control and a direct response to $\Omega _0$. Contrastingly, Runs 4–6 all present $\gamma \ge 1$, implying earlier wave breaking over the sandy terrace and a greater influence of water level over the surf zone in controlling sediment redistribution over the beach face slope.

At last, it can be observed that for Run 1 and Run 4, the beach accretion/erosion pattern is not in line with the change in $\Omega _0$, as accretion is associated with $\Delta \Omega _0 > 0$ and erosion with $\Delta \Omega _0 < 0$. This apparent inconsistency can be explained by looking at the energy(or, similarly, energy fluxes) associated with wave regimes (Table 2), which results in accretion when $\Delta E_w < 0$, and erosion when $\Delta E_w > 0$.

**Table 2:** Run descriptions.

Run 1	Run 2	Run 3
$$h_t = 5 \, \text {cm}$$		
Wr1 $\rightarrow$ Wr2 $\rightarrow$ Wr1	Wr2 $\rightarrow$ Wr3 $\rightarrow$ Wr2	Wr3 $\rightarrow$ Wr4 $\rightarrow$ Wr3
$\Omega _0$: 1 $\rightarrow$ 1.4 $\rightarrow$ 1	$\Omega _0$: 1.4 $\rightarrow$ 2 $\rightarrow$ 1.4	$\Omega _0$: 2 $\rightarrow$ 2.6 $\rightarrow$ 2
$E_{\text {w}}$: 0.49 $\rightarrow$ 0.44 $\rightarrow$ 0.49	$E_{\text {w}}$: 0.44 $\rightarrow$ 0.64 $\rightarrow$ 0.44	$E_{\text {w}}$: 0.64 $\rightarrow$ 1.33 $\rightarrow$ 0.64

Run 4	Run 5	Run 6
$$h_t = 4 \, \text {cm}$$		
Wr1 $\rightarrow$ Wr2 $\rightarrow$ Wr1	Wr2 $\rightarrow$ Wr3 $\rightarrow$ Wr2	Wr3 $\rightarrow$ Wr4 $\rightarrow$ Wr3
$\Omega _0$: 1 $\rightarrow$ 1.4 $\rightarrow$ 1	$\Omega _0$: 1.4 $\rightarrow$ 2 $\rightarrow$ 1.4	$\Omega _0$: 2 $\rightarrow$ 2.6 $\rightarrow$ 2
$E_{\text {w}}$: 0.49 $\rightarrow$ 0.44 $\rightarrow$ 0.49	$E_{\text {w}}$: 0.44 $\rightarrow$ 0.64 $\rightarrow$ 0.44	$E_{\text {w}}$: 0.64 $\rightarrow$ 1.33 $\rightarrow$ 0.64

The equilibrium beach profile associated with the first regime $Wr_n$ of each run is first reached. At $t=0$, the second wave regime $Wr_{n+1}$ is started on the previous equilibrium beach for $20\,$min. At $t=20\,$min, the beach is exposed to the $Wr_n$ for $20\,$min again. Wave regimes are defined in Table 3. Both series are performed twice for two different water levels on the terrace $h_t$.

### Discussion

**Fig. 8:**
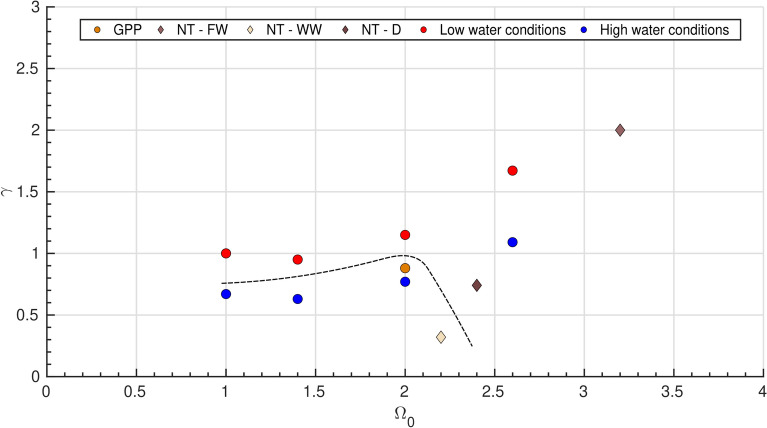
Dimensionless parameter $\gamma$ against $\Omega _0$ Experimental and field data are shown together. Scatters below the dashed black line (illustrative only) fall within Mode 1, while scatters above the dashed black line fall within Mode 2. The qualitative threshold identified in the laboratory ($\gamma$ close to unity) explains the behavior of GPP, NT–WW, and NT–FW.

The collection of these laboratory-controlled experiments successfully reproduces the two coevolution modes observed during field-measured nearshore transients. Under high water level conditions, the $\Omega _T$ threshold defined by Mingo et al.^16^ for LTT, wave-dominated environments appears to govern the switch from Mode 1 to Mode 2. However, as observed at Nha Trang, Mode 2 can also emerge under SwRB conditions during the transient period marking the start of the dry season where surface wind direction shifts, triggering the onset of a coastal upwelling. During this seasonal transition, negative SLA are recorded, and when combined with wave setup to estimate the TWLa, the data reveal that this period corresponds to a minimum in coastal water levels–distinctly lower than during the rest of the year (Fig. 5C). Laboratory simulations of low water level conditions associated to $\gamma$ $\ge$ 1 allow Mode 2 to emerge even under SwRB regimes, suggesting that nearshore water level plays a key role in controlling swash zone transients by modulating sediment redistribution between the swash and surf part of the beach profile. Indeed, water level modulation inevitably affects surf-zone morphodynamics through the emergence of local Shields and Rouse numbers associated with altered hydro-sedimentary processes, as waves transform and interact differently with the sandy bed depending on water depth^30,31^. In turn, water level modulates wave run-up within the swash zone, thereby controlling the preferential location of sediment redistribution along the beach face slope^32,33^. These results can be discussed in light of Fig. 8 where $\gamma$ values for both field and experimental data are scattered against their respective $\Omega _0$ values.

Above the dashed black line, experimental points cluster within Mode 2 (Runs 4–6 and the SRB part of Run 3), whereas those below the line (Runs 1–2 and the SwRB part of Run 3) correspond to Mode 1. The transition from Mode 1 to Mode 2 emerges around $\gamma \sim 1$ for SwRB regime. Comparison with field data supports this classification: Grand Popo lies below the threshold and follows Mode 1, consistent with expectations. A similar coherence is observed for NT–WW (Mode 1 - SwRB) and NT–FW (Mode 2 - SRB). However, the boundary between modes is less well defined for NT–D (Mode 2 – SwRB) compared to GPP (Mode 1 – SwRB) as $\gamma _{GPP} > \gamma _{NT-D}$. In this case, $\gamma$ and $\Omega _0$ alone do not provide a sufficient criterion for discrimination. This suggests that transition thresholds between Mode 1 and Mode 2 in LTT environments may be site specific. For instance, GPP and NT–WW exhibit similar modal behavior despite markedly different $\gamma$ values. A plausible explanation for these discrepancies lies in wave steepness ($s_0$), which differs substantially between the two sites: swell-dominated conditions at Grand Popo ($s_0 = 0.012$) versus wind-wave conditions at Nha Trang ($s_0 = 0.034$–0.04). Such differences may condition the site-specific $\gamma$ threshold controlling the Mode 1–Mode 2 transition. Further investigation is required to assess whether $s_0$ is a robust explanatory parameter or whether additional processes contribute to this inter-site variability.

While the laboratory model presents several limitations discussed in detail in the Methods section, the convergence of evidence from both field observations and controlled experiments suggests that water level modulations can significantly influence beach dynamics. Specifically, they appear to affect the transients (and thus the pathways) between equilibrium states, at least in LTT environments. Therefore, this study comes in addition to other laboratory experiments observing that the pathways of the EBC are not straightforward^5,34^, as the Dean number is not the only parameter of interest when studying beach transients, but here water level is too. This phenomenon introduces an additional layer of complexity to nearshore morphodynamics, warranting further investigation through both field-based analyses and experiments in wider wave flumes with reduced geometric distortion.

These results challenge the distinction between local and regional studies of beach morphodynamics, as Fig. 5A,B highlights the regional influence of upwelling on coastal water levels, leaving open questions on the broader impact of coastal processes on beach dynamics at the regional scale. Moreover, this opens new perspectives on the influence of other coastal processes capable of disrupting water levels across a wide range of timescales–from hours (e.g., transient local low-pressure systems), to days (e.g., freshwater plumes), to longer quasi-periodic scales (e.g., mesoscale coastal eddies, climate modes). This is especially important as previous field observations have shown that positive SLAs occurring at intra-seasonal scales, driven by Gulf Stream slowing, coastal downwelling, and large-scale climate oscillations, can produce erosion as significant as that caused by storm impacts^35^, while evidence from low-tide terrace beaches demonstrates that intra-seasonal SLA also modulates sediment partitioning: positive anomalies enhance upper beach erosion and terrace accretion, whereas negative anomalies favor upper beach recovery. This morphodynamic response is phase-locked with the incident wave climate, as higher water levels reduce wave dissipation over the terrace and lower levels enhance it, producing a cyclic erosion–accretion pattern with a short lag between terrace and upper beach adjustments^36^. These processes contribute to the diversity of beach responses beyond the traditional wave–tide interaction framework.

## Methods

### Field video monitoring and data validation

**Fig. 9:**
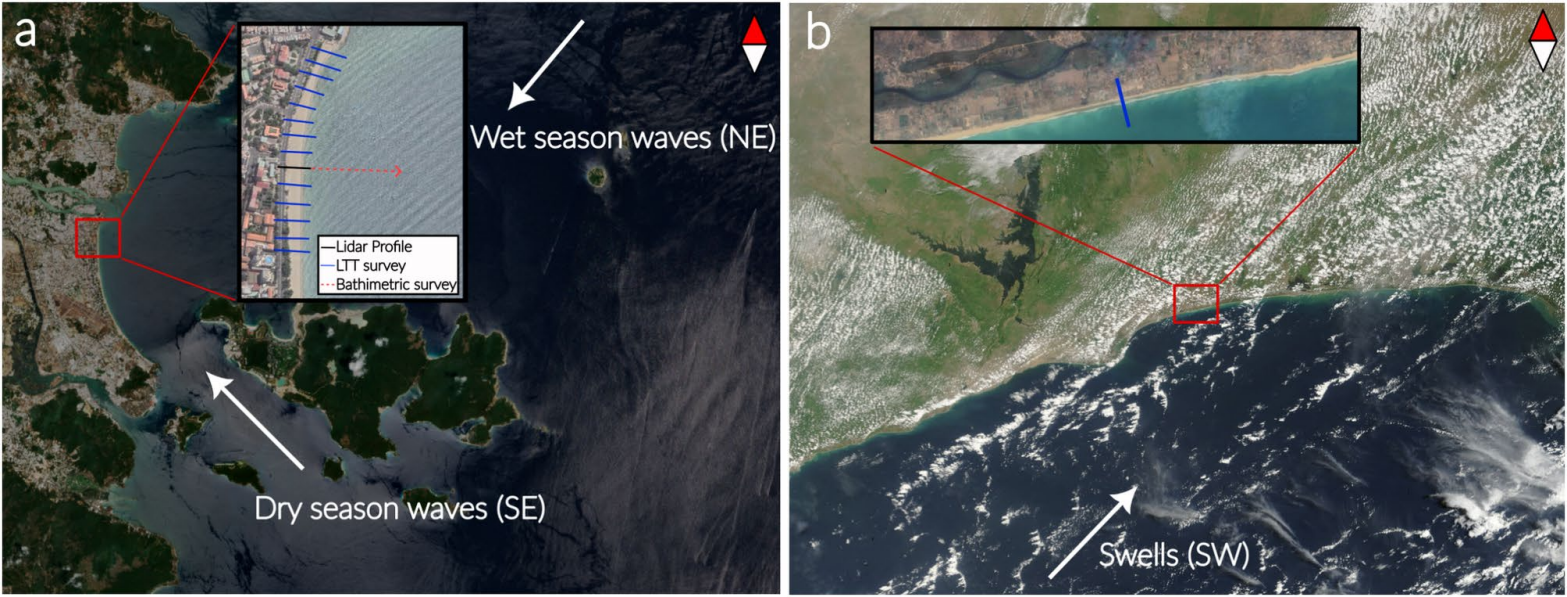
Ground-based truth sites overview (**a**) Nha Trang Bay satellite view. Insert show the specific area of beach video monitoring, along with the transect location of the LiDAR and bathymetric surveys conducted during the field campaign for validating the video-derived dataset^39^. (**b**) Grand Popo satellite view. Insert show the specific area of beach video monitoring, along with the transect location of the bathymetric surveys (using both DGPS and bathymetric sonar) conducted during the field campaign for validating the video-derived dataset^40^. Imagery © 2023 CNES/Airbus, Map data © 2023 Google.

For both beaches, the cross-shore profile generation from video monitoring is twofold: the intertidal profile is an average, over approximately 200 m alongshore, calculated by detecting the waterline at different tidal levels on time-averaged images (timex), thus smoothing irregularities such as beach cusps (L$\sim$30 m), and the subtidal profile is calculated from wave inversion techniques, on a single cross-shore time stack, in a lower terrace area pretty much alongshore uniform. The latter method using bathymetry inversion to retrieve subtidal beach profiles has now been used for more than two decades^37^, with associated root mean square error (RMSE) increasing with the distance to the shore, and ranging from 0.1 to 2 m. For both sites, RMSE in elevation (0.14 m at Grand Popo and 0.26 m at Nha Trang) were estimated by comparing inversion-method profiles with the ones observed during field surveys using both lidar derived data, topographic and bathymetric morphological surveys with differential GPS and bathymetric sonar (Fig. 9). Significant wave height and peak periods were obtained at the breaker using pixel intensity threshold^38^. For both beaches, the intensity of breaking pixels was significantly larger (Ipix > 80) than that of non-breaking pixels (Ipix $\sim$ 10). Missing hydrodynamics data due to technical video malfunction were estimated with linear regression between existing video data and Wavewatch III model version 4.10 (Grand Popo) and between existing video data and ERA-Interim global reanalysis (Nha Trang). Finally, wave setup and other sea level components such as storm surge are not included, as their estimation requires additional observations that are unavailable at these remote sites. However, while these factors can be significant over short event timescales, their influence is largely dampened over the monthly and seasonal evolution considered in this study.

A complete and tangible description of the data generation and validation can be found in Thuan et al.,^21^, Almeida et al.,^39^ and Abessolo et al.,^40^.

It should nevertheless be acknowledged that bathymetric inversion techniques are intrinsically less reliable in very shallow water and in strongly non-linear post-breaking regions, particularly close to the shoreline. In the present study, this limitation is mitigated by the hybrid construction of beach profiles, in which the intertidal portion is independently reconstructed from shoreline detection at multiple tidal levels using time-averaged imagery, while inversion is primarily applied to the subtidal and lower nearshore zone where wave transformation remains sufficiently linear. Moreover, the analysis does not rely on absolute elevations or small-scale bathymetric features in the post-breaking region, but on integrated and temporally averaged indicators of nearshore morphology, namely the beachface slope $\beta$) and the shoreline position anomaly (*Sl*), computed over spatial and temporal scales larger than the expected inversion noise. Sensitivity analyses based on Monte Carlo perturbations of profile elevations further show that the observed trends and co-evolution patterns are robust to plausible measurement uncertainties (Methods).

A second limitation of the video-derived profiles is that the intertidal component is alongshore-averaged over $\sim$200 m and the subtidal component is extracted from a single cross-shore transect assumed to be alongshore-uniform. This methodological choice necessarily filters out two-dimensional processes and alongshore variability (e.g., rhythmic morphology, alongshore gradients in sediment transport, or obliquely driven dynamics) that may influence nearshore morphodynamics. Here, however, the objective is not to resolve such bi-dimensional features, but to isolate the dominant cross-shore adjustments of the swash–surf system during transient periods: the alongshore averaging is used to suppress small-scale variability and measurement noise, thereby emphasizing coherent and persistent cross-shore signals. While alongshore processes may locally modulate sediment redistribution, the convergence of field observations and independent laboratory experiments nevertheless indicates that the identified transient behavior is primarily governed by first-order cross-shore processes, within the scope of the present dataset.

### Classifying study sites as Low Tide Terrace beaches

The equilibrium beach classification of Nha Trang and Grand Popo is now well established in the literature and is supported by a series of field studies confirming that their morphodynamics are characteristic of LTT environments^16,39,41–46^. However, it is noteworthy that Nha Trang is exposed to a wide range of wave forcing, occasionally exceeding the typical $\Omega _0$ values associated with LTT environments in the EBC framework^1,2^. In particular, during the monsoon (wet) season, Nha Trang is frequently impacted by storms, including cyclonic events, which can raise $\Omega _0$ values up to 12 (Fig. 2e). Nonetheless, the microtidal EBC is based on long-term (often seasonal) morphodynamic behavior. In this context, Nha Trang is never exposed to $\overline{\Omega _0}$ values greater than 3.5, even during the core of the wet season, and typically exhibits $\overline{\Omega _0} \sim 2.5$ throughout the rest of the year. Field analysis by Mingo et al.^16^ further showed that, even during the wet season, the averaged beach profile at Nha Trang does not transition toward a more dissipative intermediate state (Fig. 2d).

Therefore, it is reasonable to classify Nha Trang as an LTT beach, at least on a seasonal timescale.

### Defining transient events

**Fig. 10:**
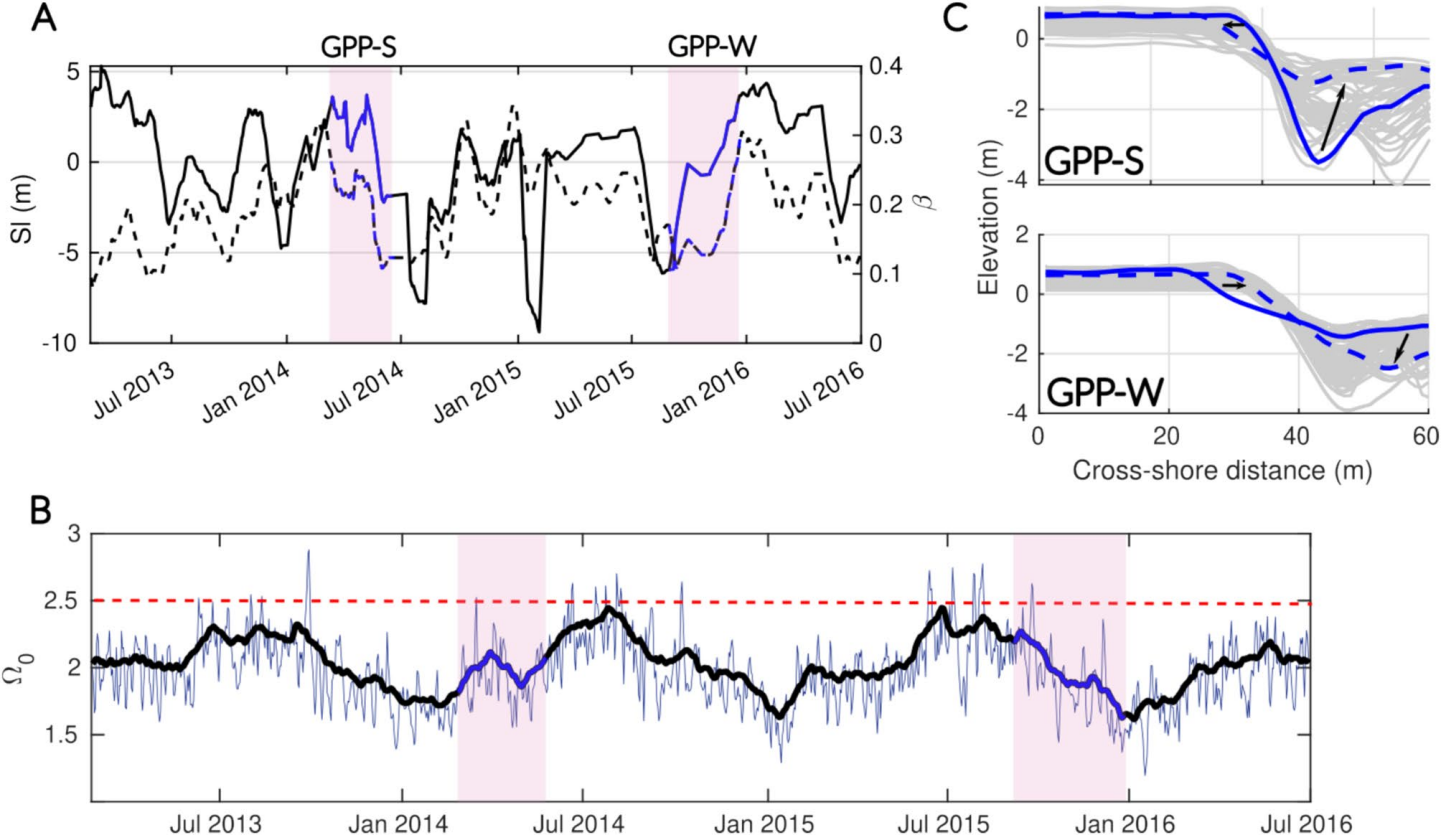
Intra-seasonal dynamics of the cross-shore profile at Grand Popo. (**a**) The evolution of *Sl* (solid line) and $\beta$ (dotted line) with time is shown with colors corresponding to the annually recurring transitions of interest (highlighted by light pink panels). Only two transitions, which oscillate seasonally between two equilibrium states and repeat themselves each year (GPP-S and GPP-W), are displayed for simplicity. (**b**) The daily $\Omega _0$ time series (blue line) is shown with its monthly averaged values (black line). The dashed red line corresponds to the $\Omega _0$ transition value ($\Omega _T$ $\sim$ 2.5) between SwRB and SRB as defined by Mingo et al.,^16^. The periods associated with the transitions are indicated by color code between the light gray panels. (**c**) The initial profile (solid line) and the final profile (dashed line) are shown with the daily transient profiles (solid gray) for each transition.

**Fig. 11:**
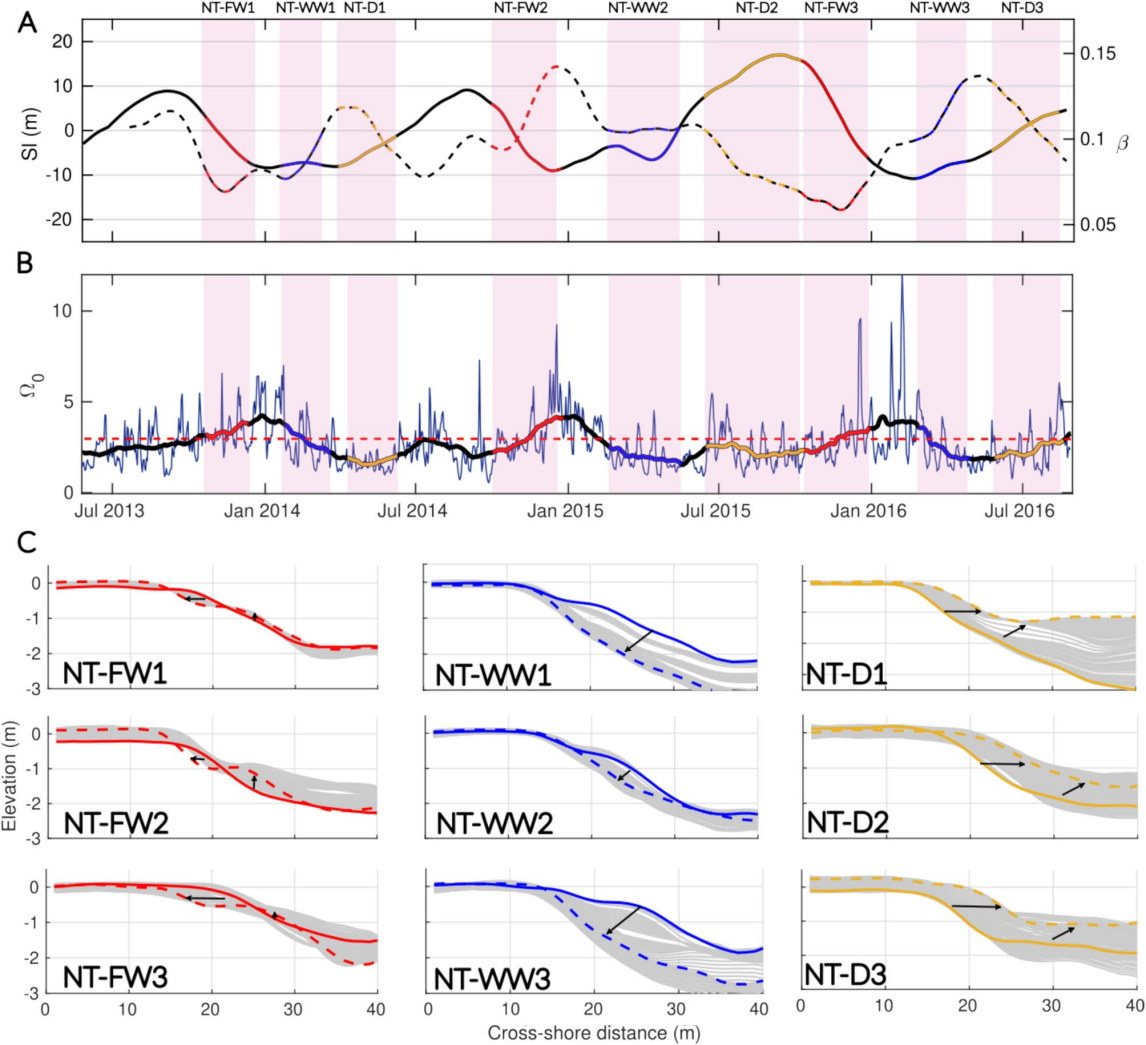
Intra-seasonal dynamics of the cross-shore profile at Nha Trang. (**a**) The evolution of *Sl* (solid line) and $\beta$ (dotted line) with time is shown with colors corresponding to the annually recurring transitions of interest (highlighted by light pink panels). (**b**) The daily $\Omega _0$ time series (blue line) is shown with its monthly averaged values (black line). The dashed red line corresponds to the $\Omega _0$ transition value ($\Omega _T$ $\sim$ 2.5) between SwRB and SRB as defined by Mingo et al.,^16^. The periods associated with the transitions are indicated by color code between the light gray panels. (**c**) The initial profile (solid line) and the final profile (dashed line) are shown with the daily transient profiles (solid gray) for each transition (NT-FW, NT-WW and NT-D) and for the three years of monitoring (1, 2 and 3).

Transients are defined as periods marked by significant morphological changes and variations in wave forcing occurring between well-identified and stationary equilibrium profiles. Based on this definition, two transients associated with seasonal shifts in wave forcing were identified at Grand Popo, while three were flagged at Nha Trang: one during the transition from dry to wet season, one during the shift from wet to dry season, and one during the latter part of the wet season, when wave energy substantially decreases. To provide a comprehensive view of these transients, the 30-day moving averages of $\Omega _0$, *Sl*, and $\beta$ are presented in Figs. 10a,b and 11a,b, along with corresponding beach profiles (Figs. 10c and 11c) for each transient period of interest.

At Grand Popo, beach states consistently fall within the SwRB regime, with $\beta$ and *Sl* following the EBC state transitions approximately 80$\%$ of the time at monthly scales. Driven by a seasonal oscillation, accretion is associated with slope steepening ($\Delta \beta > 0$ and $\Delta Sl > 0$) during the downstate transition associated with $\Delta \Omega _0 > 0$ (labeled GPP-W in Fig. 10), while erosion corresponds to slope flattening ($\Delta \beta < 0$ and $\Delta Sl < 0$) during the upstate transition associated with $\Delta \Omega _0 < 0$ (labeled GGP-S in Fig. 10).

At Nha Trang, during the three years of recorded data–labeled 1, 2 and 3–three distinct transitions recur annually (Fig. 11). The coevolution between $\beta$ and *Sl* does not consistently follow the EBC state transitions, aligning only 40$\%$ of the time at monthly scales. Two of the three identified transient periods (NT-FW and NT-D) deviate from EBC predictions (see Table 1). Over a full annual cycle, the first transition corresponds to the onset of the wet season (Fig. 11, NT-FW transition, red color). Each year, this period is marked by a steepening of $\beta$ and shoreline retreat ($\Delta \beta > 0$, $\Delta Sl < 0$). Around October, wave conditions exceed the $\Omega _T$ threshold, causing a regime shift from SwRB to SRB, which leads to rapid beach erosion and an extension of the surf zone dissipation length. The second transient occurs during the second half of the wet season. From January onward, swell energy gradually decreases (indicating a shift from SRB back to SwRB), and the beach profile deflates rapidly, with the sandy terrace disappearing. During this time, *Sl* alternates between accretive and erosive phases, while $\beta$ varies continuously until March or April, when the reflective winter profile becomes fully established (Fig. 5, NT-WW transition, blue color). On average, over the entire transient, *Sl* advances and $\beta$ steepens. Finally, the third transient occurs during the transition from the wet to the dry season (Fig. 11, NT-D transition, orange color). The gentler summer swell (SwRB regime) promotes significant beach accretion and the gradual formation of a low, submerged sandy terrace, while the beach face slope flattens ($\Delta \beta < 0$, $\Delta Sl > 0$).

### Uncertainties on slope estimation

**Fig. 12:**
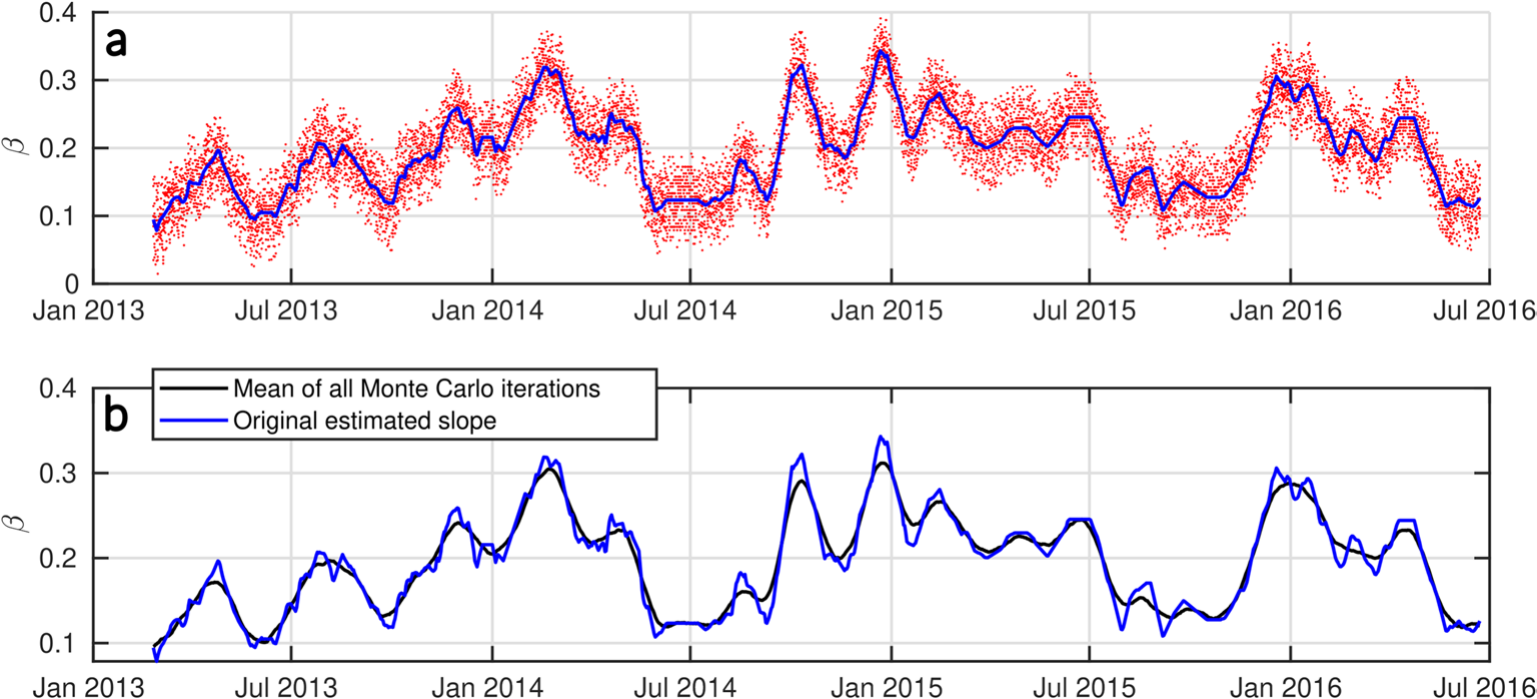
Monte Carlo test on the estimated profile position error at Grand Popo. (**a**) Each iteration of the Monte Carlo (red dots) and the original estimated slope (solid blue). (**b**) Mean of all the Monte Carlo iterations (solid black) and the original estimated slope (solid blue).

**Fig. 13:**
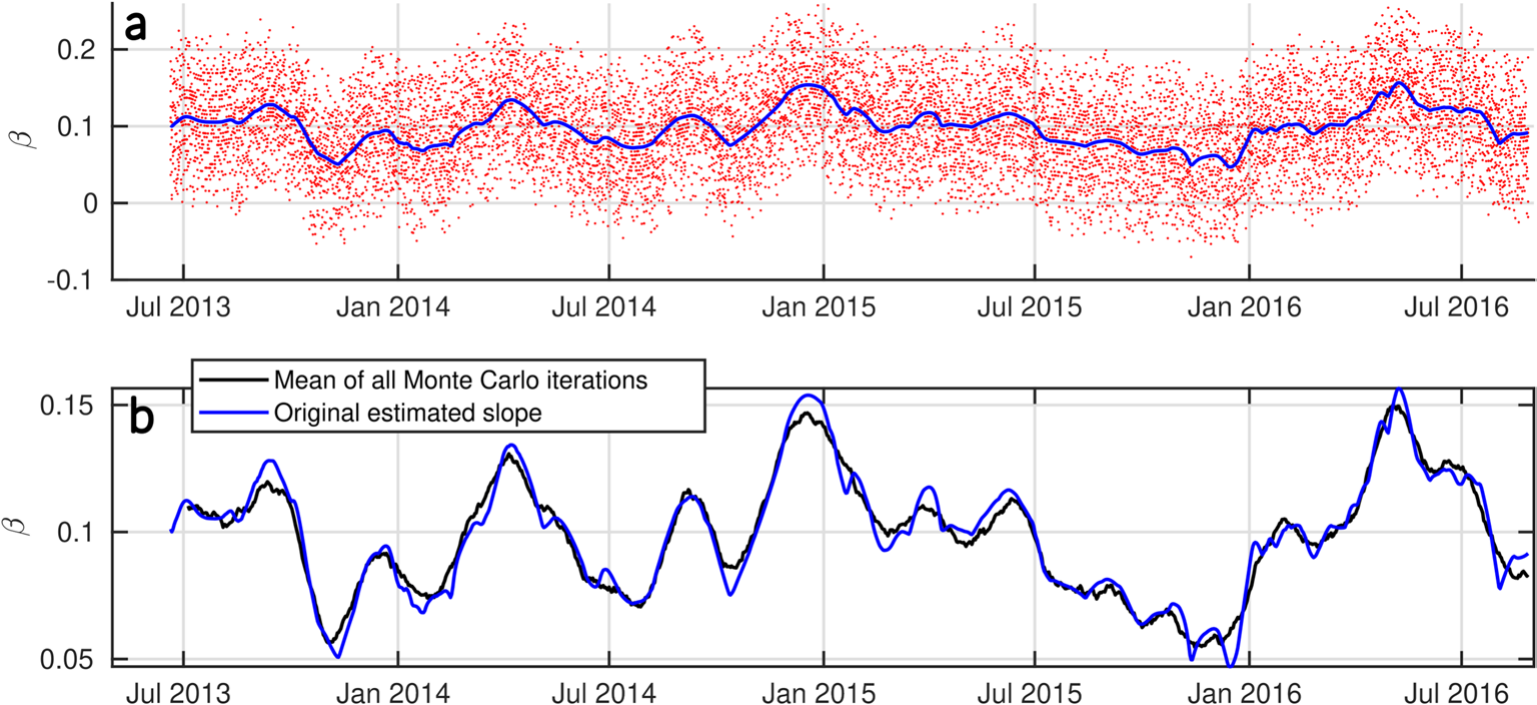
Monte Carlo test on the estimated profile position error at Nha Trang. (**a**) Each iteration of the Monte Carlo (red dots) and the original estimated slope (solid blue). (**b**) Mean of all the Monte Carlo iterations (solid black) and the original estimated slope (solid blue).

In order to give some more insights into the slope errors based on elevation uncertainties, we have conducted a Monte Carlo test to assess error propagation in the estimation of the slopes. Since $\beta$ is measured between the upper and lower parts of the swash slope^16^, we shifted both ends of the slope within a range from -(RMSE/2) to +(RMSE/2) values specific to each site (by step of 0.01 m). The Monte Carlo method allows for different errors to interact across iterations, generating a time series of swash slope estimates with varying errors at both ends. Each time series of the newly estimated $\beta$ represents the sensitivity of slope estimation to potential errors in profile position measurements. The results of this analysis are presented in Figs. 12 and  13, showing that while extrema and small anomalies can be slightly (but not significantly) altered, the overall trend and the slope values are preserved within the range of errors tested.

### Water level estimations

Here, the proper estimation of coastal water level fluctuations requires accounting for two key phenomena:

- **Wave setup**: Generated by wave breaking and bore propagation in the nearshore zone, wave setup influences water levels from the shoreline up to the breakpoint, as demonstrated by field observations^47^. At Nha Trang, wave setup is estimated using the empirical formulation from Stockdon et al.^29^, given by $\eta = 0.35\beta \sqrt{H_s\lambda _0}$, where $\beta$ is the daily beach face slope derived from video imagery.
- **Sea level anomaly**: Defined as the deviation from the long-term regional average (based on a 5-month running mean), SLA reflects the combined influence of oceanographic and atmospheric processes. These include (non-exhaustive) tides, coastal upwelling, barotropic responses, and geostrophic adjustments, each acting over different timescales and frequencies. SLA data are obtained from satellite altimeter observations provided by the European Center for Medium-Range Weather Forecasts.

By combining $\eta$ and SLA, we estimate the total water level anomaly (TWLa), which serves as a first-order proxy for tracking coastal water level variations over time.

### Physical based model

#### Laboratory setup

Assuming the dominant influence of wave forcing on the upper beach profile, an idealized wave flume model was developed to replicate the key characteristics of the reference sites on a laboratory scale. The primary objective of this laboratory model is to investigate the types of transitions illustrated in the Swash Dynamic Diagram (Fig. 1) and to characterize the associated processes, offering a physical framework to interpret field observations, through qualitative confrontations between both scales.

**Fig. 14:**
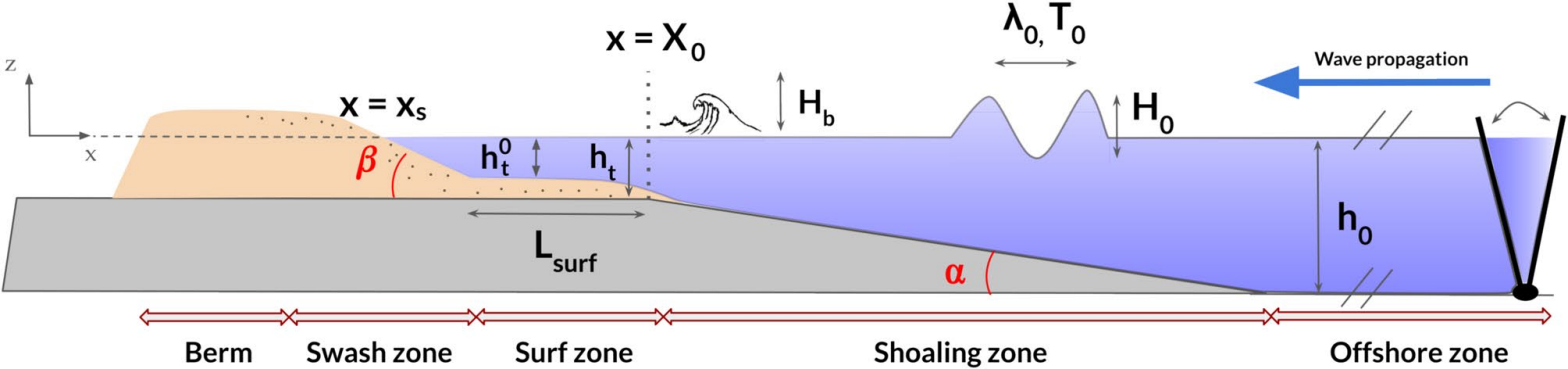
Technical scheme of the laboratory setup. The initial erodible sandy beach profile is built from the berm to the breaker zone. The monochromatic wave forcing breakpoint is controlled by the slope break at the beginning of the surf zone ($X_0$). Offshore water level, terrace water level and wave regime are all control parameters of the experiments. Arrows outlined in red indicate the experimentally defined zones. In the physical based model, the nearshore includes both the swash and the surf zone.

The experiments consist of controlled wave forcing incoming a nearshore model partly composed of erodible sand, which were conducted in a unidirectional wave channel of $14\,$ m length, $0.3\,$ m height and $0.14\,$ m width (Fig. 14). The designed model aims to create rapid upper beach equilibria and study the mechanism of transient events. For this purpose, monochromatic waves are generated by a flap paddle at one end of the channel. Recent studies examining cross-shore profile evolution toward equilibrium for different wave types (monochromatic, bichromatic, random), indicate that monochromatic waves tend to produce morphological evolutions similar to those generated by other wave types^48^. Furthermore, sediment transport in polychromatic wave conditions is predominantly driven by the largest waves within the groups, making the significant wave height used in the Dean number definition the primary factor influencing sediment transport^49^. Consequently, given that low-tide terrace beaches are dominated by gravity waves^50^, the application of the present model (which utilizes an idealized nearshore geometry scaled with the offshore Dean number (Table 5)) did not reveal any unexpected morphological features.

At the other end of the flume, a rigid, non-erodible slope ($\alpha$ = 0.06) is used to impose an artificial shoaling zone, allowing to mostly control the position of the breakpoint at $x=X_0$, with only small variations around it. This setup can be used to simulate LTT with rocky foreshore as in Mingo et al.,^17^, but can also provide a rigid layout for the construction of a fully developed sandy beach, from the breakpoint to the berm, in order to allow sediment exchange between laboratory swash and surf zones. The sediment used here consists of fine quartz grains of high density ($\rho _{s}$ = 2650 kg $\cdot$ $m^{-3}$) and with a mean size of $D_{50}$ = 0.12 mm, corresponding to a measured settling velocity of $w_s = 0.0074\,$m $\cdot$ s$^{-1}$. Wave propagation is monitored at a frequency of 500 Hz by a series of wave gauges placed every two meters in the offshore zone. Using a light attenuation technique (backlighting of the water tank), the morphological evolution of the beach and the hydrodynamic transformation are recorded over a channel length of 0.9 m with a high-resolution sCMOS-Lavision camera (field width of 2300 pixels and error on the cross-shore profile position estimation of 9 × $10^{-4}$ m) at a frequency of 0.02 Hz and 60 Hz respectively.

The offshore water level in the flume is kept constant during the experiments $h_0$ = {0.16, 0.17  m} and is associated with two possible water level configurations over the terrace. The wave regimes ($H_0$, $T_0$), offshore wave height and period are characterized at the beginning of the rigid slope by a wave gauge, just before entering the shoaling zone (Table 3). The latter is characterized by a constant angle $\alpha$. The surf zone, $x<X_0$, is initially covered with 2 cm of sand. This zone evolves with sediment transfer and is characterized by a dissipation length called $L_{surf}$, which is initially set to 0.25 m. This length is estimated between the foot of the linear beach face slope and the mobile breakpoint $x = X(t)\approx X_0$, which can be slightly displaced by wave action during the experiments. As a result, the absolute water elevation above the flat, rigid base is used as a reference, and the initial terrace water level $h^0_t = h_t(0)$ is employed to estimate dimensionless scaling parameters. Two initial water levels $h_t$ were tested in the experimental series: $h_t = 0.04$ m (corresponding to $h_0 = 0.16$ m and $h^0_t = 0.02$ m) and $h_t = 0.05$ m (i.e., $h_0 = 0.17$ m and $h^0_t = 0.03$ m). Finally, the swash zone subject to bore surge during the experiments is composed entirely of sand.

As for field data, the swash zone is characterized by its beach face slope angle $\beta$ and the shoreline anomaly (*Sl* = $x_s(t)-S$). $S=\overline{x_s(t)}$ is the averaged shoreline position over the entire experiment and $x_s(t)$ is estimated at the steady state water level in the absence of waves.

**Table 3:** Laboratory hydrodynamic parameters at the beginning of the shoaling zone.

Wave Regime	$T_{0}$ (s)	$\sigma _{T_p}$ (s)	$H_{0}$ (m)	$\sigma _{H_0}$ (m)	$\lambda _0$ (m)	$E_{waves}$ (J/m^2^)	$E_{Flux}$ (m^2^ s)	$\Omega _{0}$
Wr1	2.55	3.7 $\times 10^{-4}$	0.020	4.8 $\times 10^{-4}$	3.1	$0.49 \pm 0.02$	$(1.02 \pm 0.05)\times 10^{-3}$	$1.00 \pm 0.02$
Wr2	1.80	6.6 $\times 10^{-2}$	0.019	1.0 $\times 10^{-3}$	2.2	$0.44 \pm 0.05$	$(6.51 \pm 0.70)\times 10^{-4}$	$1.40 \pm 0.07$
Wr3	1.53	1.4 $\times 10^{-4}$	0.023	4.3 $\times 10^{-4}$	1.8	$0.65 \pm 0.02$	$(8.10 \pm 0.3)\times 10^{-4}$	$2.00 \pm 0.04$
Wr4	1.65	1.5 $\times 10^{-2}$	0.033	6.0 $\times 10^{-4}$	2.0	$1.33 \pm 0.05$	$(1.85 \pm 0.07)\times 10^{-3}$	$2.60 \pm 0.05$

Wave regimes (Wr) associated Dean number values increase from Wr1 to Wr4. Wave gauges precision was inferred by computing the standard deviation between the calibration data (every 2.5 x $10^{-3}$ m) and the fitted linear law and is $\sigma _{gauge}$ = 5.2 x $10^{-4}$ m. $T_p$ and $H_s$ standard deviations ($\sigma _{T_p}$, $\sigma _{H_s}$) are computed over 5 to 10 wavelengths. Errors in $E_{waves}$, $E_{Flux}$ and $\Omega _0$ are computed using the uncertainty propagation method.

### Dimensionless analysis

Building a downscaled physical model of a complex natural system such as sandy beaches comes with a set of physical parameters characterizing its dynamics. In the present model, these parameters are related to the sediment properties ($w_s$, $\rho _{s}$), the wave parameters ($H_0$, $T_0$), the fluid properties (density $\rho$, kinematic viscosity $\nu$), a water depth *h* and the gravitational acceleration *g*^51^. As we are focusing on the nearshore dynamics and not the whole process of beach motion from the depth of closure, we choose the water depth to be $h=h^0_t$, i.e. to scale the experiments based on a typical depth over the terrace in the surf zone. Then, to ensure the finest scaling, five dimensionless numbers must be scaled up from the field to the laboratory^52^.

A height ratio $\gamma$, a Reynolds number *Re*, the density ratio $\phi$, a nearshore Rouse number *Ro* and the Dean number $\Omega _0$, are chosen here and defined as, respectively:

1 $$\begin{aligned} Ro = \frac{w_s}{\sqrt{g h^0_t}}, \quad Re = \frac{H_0 \sqrt{g h^0_t}}{2 \nu }, \quad \phi = \frac{\rho _s}{\rho }, \end{aligned}$$

2 $$\begin{aligned} \Omega _0 = \frac{H_0}{w_s T_0}, \quad \gamma = \frac{H_0}{h^0_t} \end{aligned}$$

In (1), *Ro* represents an estimation of the efficiency to transport sediment within the surf zone. It can be noted that *Ro* is not the conventional definition of the Rouse number as found in the literature for coastal flows^51^, the latter being somewhat similar to the Dean number, designed to quantify the ability of waves to set and maintain particles in vertical suspension. Here, it represents the ability of a wave propagating in the surf zone and driven by its long wave celerity approximation to transport sand prior settling. Then the latter expresses the ratio between cross-shore motion in the surf zone and the vertical settling. Slightly lower values were found in the laboratory compared to the field. However, these values are indicative of suspended transport, confirming that the transport regime remains comparable in both cases. *Re* is impossible to scale due to the dimensions of the experiment in relation to the field. Yet, it is often assumed to be high enough to provide the required turbulent flow characteristics. For a sandy bottom layer, Jonsson et al.,^53^ assumed that the oscillatory flow due to waves reaches a fully established turbulent regime for Re > 1000, a threshold that is exceeded in the laboratory (Table 4). $\phi$ can slightly vary depending on the silicate mixture that makes up the beach. Nevertheless, this ratio is of the same order of magnitude, both in the field and in the experiments.

In (2), $\gamma$ provides an indication of the surf zone geometry relative to the incident wave height. This breaker index can be interpreted as a Froude number established in shallow water conditions, representing the balance between inertial and gravitational forces. It is important to note that, as with all movable-bed experimental models, the Froude number (e.g., $\gamma$) becomes an observable parameter once the bed profile begins to respond to the incident forcing. As such, $\gamma$ serves as an indicator of both nearshore turbulence intensity and the occurrence of wave breaking. In this study, wave breaking occur around $x = X_0$ when $\gamma > 0.7$. Furthermore, wave breaking is assumed to be dynamically scaled when the surf similarity parameter $\epsilon _a$ is also scaled appropriately^54^, as shown in Table 4. Lastly, the Dean number is associated with the microtidal EBC^1^ and characterizes the seasonal response of the beach to the wave climate. Initially defined with the bore height rather than the offshore height, the limitations associated with obtaining $H_b$ has gradually imposed the current formulation within the community as correlations between $\Omega _0$ and equilibrium states were strong enough to overlook $H_b$. Note that, according to its definition, the Dean number mostly compares vertical velocities of waves and particles. In the laboratory, its scaling ensures that the profile constructed by the different wave regimes exhibits typical nearshore characteristics depending on the Dean number value.

**Table 4:** Summary of the dimensionless numbers as defined in (1) and (2) for both field and laboratory environments.

Sites	$\gamma$	*Re*	$\phi$	*Ro*	$\Omega _{0}$	$\frac{L_{surf}}{\lambda _t}$	$\frac{h_t}{L_{surf}}$	$\epsilon _a = \frac{\alpha }{\sqrt{\frac{H_0}{\lambda _0}}}$
Laboratory	$[0.6 - 1.7]$	[$10^3$–$10^4$]	2.65	[0.017–0.014]	[1–2.6]	[0.19 -0 .36]	[0.08–0.12]	0.5–0.7
Nha Trang	$[0.32 - 1.7]$	[$10^5$–$10^6$]	2.59	[0.024–0.037]	[1.7–3.5]	0.3	0.05	-
Grand Popo	0.88	$10^6$	2.59	0.021	[1.7–2.4]	0.3	0.04	-

For field parameters, values are estimated using averaged quantities $\overline{\cdot }$. $\lambda _t$, the wavelength at the breaker ($x = X_0$), has been assessed by transforming $\lambda _0$ according to linear wave theory in intermediate water conditions. $\alpha$ corresponds to the rigid shoaling slope.

Ensuring that the values of the dimensionless numbers defined in Eqs. (1) and (2), fall within the ranges found in the field and that their physical interpretations correspond to reality ensure the best scaling possible for our laboratory-scaled physical model. Note that it is possible to define the dimensionless numbers differently^51,55^. The choice mostly depends on the dynamics and physics to be represented. In any case, it is possible to establish connection between them. Here, we focus on the nearshore dynamics, and more specifically on the transport and morphodynamics change in this zone. Accordingly, $\Omega _0$ and $\gamma$ are the main dimensionless numbers of interest, which are scaled between field and laboratory model.

#### Scaling ratios: coastal literature based methods

Following physical modeling theory^56^ and based on coastal modeling literature, it is possible to examine the scaling relationship between the laboratory model and the field prototype to draw similar conclusions as the ones from the previous Subsection. This is achieved based on linear wave theory^57^ by defining a set of scaling ratios, each reflecting a different physical parameter of interest:

- Wave height scale ($N_h$)
- Temporal scale ($$N_t$$)
- Mobility scale ($$N_M$$)
- Wave period scale ($N_T$)
- Length scale ($$N_L$$)
- Height scale ($$N_H$$)
- Orbital velocity scale ($N_u$)
- Wavelength scale ($$N_{\lambda }$$)
- Water level scale ($N_{h_t}$)

These ratios are defined as:

3 $$\begin{aligned} N_h = \frac{H_s^{p}}{H_s^{m}}, \quad N_t = \sqrt{N_h}, \quad N_M = \frac{w_s^{p}}{w_s^{m}}, \quad N_T = \frac{T^p_p}{T^m_p}, \quad N_L = \frac{L_p}{L_m}, \quad N_H = \frac{H_p}{H_m}, \quad N_u = \frac{u_{p}}{u_{m}}, \quad N_{\lambda } = \frac{\lambda _{p}}{\lambda _{m}}, \quad N_{h_t} = \frac{h^p_t}{h^m_t} \end{aligned}$$

where $L_p$, $H_p$ and $L_m$, $H_m$ represent the typical beach length and height in the prototype and the model, respectively^54,58,59^.

Based on field observations and considering the study’s focus on nearshore beach dynamics, a representative surf zone length in the field is taken as $L_p \approx 10$ m, and the typical swash–surf vertical scale is $H_p \approx 2$ m. In the laboratory model, $L_m = 0.2$ m and $H_m = 0.1$ m (as described in the previous subsection). This yields a geometric distortion ratio of $N_g = \frac{N_L}{N_H} = 2.5$. According to Noda et al.^58^, acceptable prototype–model distortion values fall within the range of 1 to $(N_H)^{0.32}$. For the present setup, this upper limit equals 2.61. Therefore, although the model is notably distorted, it remains within the generally accepted range for qualitative beach modeling studies.

This assertion holds only if Froude similarity is preserved:

4 $$\begin{aligned} N_h = N_{\lambda } = N_T^2 = N_{h_t} = N_u^2 \end{aligned}$$

In the present laboratory setup, all physical quantities have been adjusted to satisfy unity in (2) (see Table 5 for the complete set of scaling values used). As a result, Froude similarity is respected between the model and the prototype. The latter implies that the undertow is scaled^54^.

The choice of $D_{50}$ (and thus $w_s$) at the laboratory scale (based on the range of generated wave heights and the need to reproduce sediment transport modes from bedload to suspended load) constrains the mobility ratio to approximately 10. This means that the sand size does not satisfy the length ratio of $\approx 100$ between field and laboratory, indicating that the grains are larger relative to the wave height in the laboratory compared to the field. Nevertheless, this choice allows the experimental model to reproduce the relevant physical transport mechanisms during bore propagation in the nearshore; however, it may lack other second-order mechanisms that are not of interest in the present study, such as infiltration processes in the beach. Finally, the temporal scaling ratio is constrained by the wave, mobility, and length scales, resulting in a time scale ratio of $N_t \approx 10$.

**Table 5:** Table of the scaling ratios’ order of magnitude.

Physical quantity	Laboratory	Grand Popo	Nha Trang
$H_s$ (m)	0.01	1	1
$\mathbf {N_h}$	**100**		
$\lambda$ (m)	1	100	[10–50]
$\mathbf {N_\lambda }$	**100**		
$T_p$ (s)	1	10	10
$\mathbf {N^2_T}$	**100**		
$h_t$ (m)	0.01	1	1
$\mathbf {N_{h_t}}$	**100**		
*u* ($m\,\text {s}^{-1}$)	0.3	3	3
$\mathbf {N^2_u}$	**100**		

Recap of the physical quantities extracted from field data and laboratory controlled environments to establish Froude similarity.

#### Potential limitations of the flume geometry

Given the small geometric scale of the experiment, the potential influence of surface tension was assessed and ruled out based on two key estimations:

- **Capillary length via the Bond number:** The Bond number, defined as $Bo = \frac{\rho gL^2}{\sigma }$, compares gravitational to surface tension forces. Assuming the limiting case of $Bo = 1$, the corresponding capillary length for freshwater is approximately 2.7 mm. As the water depth in the nearshore exceeds this value, capillary effects are negligible, allowing for the development of turbulent flow.
- **Weber number:** The Weber number, $We = \frac{\rho v^2 L}{\sigma }$, compares fluid inertial forces to surface tension forces. The analysis yielded $We \approx 10^3 \gg 1$, indicating that inertial forces strongly dominate over surface tension effects in the experimental setup.

These results confirm that surface tension does not significantly influence the flow, validating the suitability of the experimental configuration for investigating sediment transport processes.

The influence of containment effects in a narrow flume setup is twofold: (1) sidewalls can increase energy dissipation through friction, and (2) they may induce circulation cells that disrupt sediment transport in an unnatural way. Regarding (1), wave gauge measurements along the full length of the flume (up to the breaking point) indicate that sidewall friction does not result in significant wave energy dissipation. For (2), the horizontal uniformity observed across iso-elevations of the beach profile suggests that any potential sidewall-induced recirculation cells are effectively disrupted by turbulence in the nearshore zone.

Moreover, the laboratory experiments presented here rely on a dynamic scaling strategy that prioritizes the preservation of the dominant dimensionless parameters governing nearshore morphodynamics rather than strict geometric similitude. Sediment grain size is treated as a reference parameter, with wave and water-level conditions adjusted to maintain realistic values of the Dean number, Rouse number, and breaker index. While exact grain-size similitude cannot be achieved without introducing additional distortions, this choice avoids shifting the system toward a different physical regime (e.g., Stokes settling or cohesive-dominated transport) and ensures that the governing sediment transport processes and feedbacks remain comparable to those observed in the field. Similarly, the experiments are restricted to normally incident waves, which does not reproduce the seasonal variability in wave direction. This choice is intentional and reflects the focus on cross-shore sediment exchanges controlling beachface slope and shoreline position. Including oblique wave incidence would introduce longshore transport processes that are beyond the scope of this study and would obscure the interpretation of the transient cross-shore pathways investigated here. The convergence of field observations and independent laboratory experiments nevertheless indicates that the identified transient behavior is primarily governed by first-order cross-shore processes.

## Data Availability

Field and laboratory data supporting the conclusions of this study are made available on Zenodo (Dataset). Sea surface temperature data are extracted from ODYSSEA Global Sea Surface Temperature Gridded Level 4 Daily Multi-Sensor Observations (https://doi.org/10.48670/mds-00321). Sea level anomaly data are extracted from Global Ocean Gridded L 4 Sea Surface Heights And Derived Variables Nrt (https://doi.org/10.48670/moi-00149). Surface wind directions and intensities are extracted from Global Ocean Physics Reanalysis (https://doi.org/10.48670/moi-00021)
